# Heat Shock Protein 27, a Novel Downstream Target of Collagen Type XI alpha 1, Synergizes with Fatty Acid Oxidation to Confer Cisplatin Resistance in Ovarian Cancer Cells

**DOI:** 10.3390/cancers13194855

**Published:** 2021-09-28

**Authors:** James Patrick Heiserman, Sameera Nallanthighal, Cody C. Gifford, Kayla Graham, Rohan Samarakoon, Chao Gao, Jessica J. Sage, Wenzheng Zhang, Paul J. Higgins, Dong-Joo Cheon

**Affiliations:** Department of Regenerative and Cancer Cell Biology, Albany Medical College, Albany, NY 12208, USA; heiserj@amc.edu (J.P.H.); nallans@amc.edu (S.N.); gifforc@amc.edu (C.C.G.); k27grah@siena.edu (K.G.); samarar@amc.edu (R.S.); gaoc@amc.edu (C.G.); sagej@sage.edu (J.J.S.); zhangw1@amc.edu (W.Z.); higginp@amc.edu (P.J.H.)

**Keywords:** COL11A1, cisplatin resistance, ovarian cancer, HSP27, FAO

## Abstract

**Simple Summary:**

Collagen type XI alpha 1 (COL11A1) is a novel biomarker associated with poor survival in ovarian cancer and a promoter of ovarian cancer cell resistance to cisplatin. However, it is poorly understood how COL11A1 promotes ovarian cancer cisplatin resistance. We performed assays to discover the biological molecules that are activated by COL11A1 in ovarian cancer cells. We found that heat shock protein 27 (HSP27), a cellular stress response protein, is activated by COL11A1. Furthermore, we observed that depletion and drug inhibition of HSP27 makes ovarian cancer cells grown on COL11A1 to be more susceptible to cisplatin treatment. We also discovered that ovarian cancer cells upregulate fatty acid oxidation (FAO), a metabolic process that breaks down fats to generate energy and biomolecules, to compensate for the loss of HSP27. Our findings have therapeutic implications for clinicians who wish to treat ovarian tumors that maintain high levels of COL11A1 and HSP27.

**Abstract:**

Collagen type XI alpha 1 (COL11A1) is a novel biomarker associated with cisplatin resistance in ovarian cancer. We have previously reported that COL11A1 activates Src-Akt signaling through the collagen receptors discoidin domain receptor 2 (DDR2) and integrin α1β1 to confer cisplatin resistance to ovarian cancer cells. To identify the potential signaling molecules downstream of COL11A1 signaling, we performed protein kinase arrays and identified heat shock protein 27 (HSP27) as a potential mediator of COL11A1-induced cisplatin resistance. Through receptor knockdown and inhibitor experiments, we demonstrated that COL11A1 significantly upregulates HSP27 phosphorylation and expression via DDR2/integrin α1β1 and Src/Akt signaling in ovarian cancer cells. Furthermore, genetic knockdown and pharmacological inhibition of HSP27, via ivermectin treatment, significantly sensitizes ovarian cancer cells cultured on COL11A1 to cisplatin treatment. HSP27 knockdown or inhibition also decreases NFκB activity as well as the expression of inhibitors of apoptosis proteins (IAPs), which are known downstream effector molecules of COL11A1 that promote cisplatin resistance. Interestingly, HSP27 knockdown or inhibition stimulates ovarian cancer cells to upregulate fatty acid oxidation (FAO) for survival and cisplatin resistance, and dual inhibition of HSP27 and FAO synergistically kills ovarian cancer cells that are cultured on COL11A1. Collectively, this study identifies HSP27 as a novel and druggable COL11A1 downstream effector molecule that may be targeted to overcome cisplatin resistance in recurrent ovarian cancer, which often overexpress COL11A1.

## 1. Introduction

Cisplatin resistance is a major challenge in the effective treatment of ovarian cancer, the most lethal gynecological cancer in the United States. Dysregulated extracellular matrix (ECM) accumulation is a predominant contributor to ovarian cancer progression and cisplatin resistance [1]. For example, the expressions of several collagen genes are upregulated in ovarian cancer and are also associated with acquisition of chemotherapy resistance [2,3,4]. Collagen type XI alpha 1 (COL11A1) encodes one of three alpha chains of type XI collagen, a minor fibrillar collagen that is crucial for collagen fiber assembly and organization [5,6]. In ovarian cancer, COL11A1 is primarily expressed by cancer-associated fibroblasts (CAFs) [7], although cisplatin-resistant ovarian cancer cells have also been shown to express COL11A1 [8,9,10]. Our group and others have demonstrated that elevated expression of COL11A1 is associated with poor survival, recurrence, and cisplatin resistance in ovarian cancer [8,9,10]. Our previous studies have demonstrated that COL11A1 confers cisplatin resistance through activation of discoidin domain receptor 2 (DDR2) and the integrin heterodimer α1β1, which in turn activates Src, Akt, and NFkB to upregulate the expression of inhibitors of apoptosis (IAPs) [8,9]. We have also discovered that COL11A1 confers cisplatin resistance through switching the metabolic preference of ovarian cancer cells to fatty acid metabolism [8]. However, it is not fully understood which signaling molecules are activated by COL11A1 to regulate these pathways leading to cisplatin resistance.

Heat shock protein 27 (HSP27), encoded by the HSPB1 gene, is a protein chaperone of the small heat shock protein group that is implicated in cancer cell survival, chemotherapy resistance, and other cancer-related processes across many cancer types, including gastric, colon, lung, and pancreatic cancers [11,12,13]. In ovarian cancer, several studies have established the relationship between HSP27 expression and poor patient survival and chemotherapy resistance [14,15,16,17,18]. Langdon et al. [16] showed that high levels of cytosolic HSP27 expression are positively correlated with advanced cancer stage and reduced patient survival. Zhao et al. [18] found that elevated HSP27 expression increases peritoneal metastasis in ovarian cancer. Elevated serum HSP27 is associated with clinical stage, ascites volume, and residual tumor mass in ovarian cancer [14,15,17]. In vitro studies have shown that HSP27 inhibition sensitizes ovarian cancer cells to paclitaxel and cisplatin [19,20]. Although these studies have implicated HSP27 in ovarian cancer progression, little is known how the tumor microenvironment, particularly ECM proteins, regulates HSP27 in progressive ovarian cancer. Given COL11A1’s involvement in signaling ovarian cancer cell cisplatin resistance, we next explored the link between COL11A1 and HSP27.

In this study, we report that COL11A1 upregulates HSP27 expression and activity through DDR2/integrin α1β1-Src-Akt signaling to induce cisplatin resistance in ovarian cancer cells. HSP27 mediates COL11A1-dependent activation of NFkB and upregulation of IAPs. Interestingly, we also discovered that fatty acid oxidation (FAO) compensates for the loss of HSP27 in ovarian cancer cells cultured on COL11A1 and dual inhibition of FAO and HSP27 is lethal to cisplatin-resistant ovarian cancer cells cultured on COL11A1. This study identifies HSP27 as a novel and druggable downstream molecule of COL11A1 to attenuate cisplatin resistance in ovarian cancer cells.

## 2. Materials and Methods

### 2.1. Cell Culture

A2780 and A2780CIS cells were purchased from SIGMA (St. Louis, MO, USA). ES2, SKOV3, and A204 cells were purchased from ATCC (Manassas, VA, USA). Lenti-X 293T cells were purchased from Takara bio (formerly Clontech, Kusatsu, Shiga, Japan). A2780 and A2780CIS cells were cultured in RPMI (Gibco Life Technologies; Waltham, MA, USA) supplemented with 10% FBS and 1× penicillin/streptomycin. ES2, SKOV3, A204, and Lenti-X 293T cell lines were cultured in DMEM (Gibco Life Technologies; Waltham, MA, USA) supplemented with 10% FBS (Sigma-Aldrich, St. Louis, MO, USA) and 1× penicillin/streptomycin (Gibco Life Technologies; Waltham, MA, USA). All cells were cultured at 37 °C with 5% CO_2_. All cell lines tested negative for mycoplasma contamination and were authenticated before experimentation.

### 2.2. Cell Culture on Collagen Extract and Acellular Matrix

**Figure 1:**
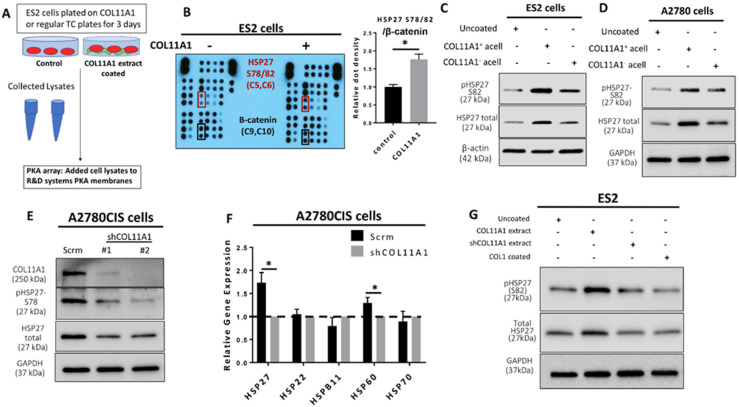
COL11A1 induces HSP27 phosphorylation and total expression in ovarian cancer cells. (**A**) Schematic of the Protein Kinase array (PKA) experiment set up. (**B**) Image and quantification of the PKA result. Red boxes on the image indicates the position of the HSP27 S78/82 antibody epitopes, and black boxes indicate the β-catenin epitopes. Coordinates for the HSP27 S78/82 epitopes and β-catenin on membranes are listed below the protein name in parentheses. Bar graph shows quantification of the HSP27 (S78/82) dot densities normalized to β-catenin. (**C**) Western blot of the phosphorylated (at S82) and total HSP27 in ES2 cultured on COL11A1-positive and -negative A204-derived decellularized matrices (referred to as acell) and control (uncoated) conditions. (**D**) Western blot of the phosphorylated (at S82) and total HSP27 in A2780 cells cultured on COL11A1-positive and -negative A204-derived decellularized matrices and control conditions. (**E**) Western blot of phosphorylated (at S78) and total HSP27 in A2780CIS-scrm and A2780CIS-shCOL11A1. (**F**) Gene expression of HSP27 (HSPB1), HSP22 (HSPB8), HSPB11, HSP60 (HSPD1), and HSP70 (HSPA1A) in A2780CIS-scrm and A2780CIS-shCOL11A1 measured by RT-PCR. (**G**) Western blot of the phosphorylated (at S82) and total HSP27 in ES2 cultured on COL11A1-positive and -negative extracts and control conditions (*n* = 3). GAPDH and β-actin were used as the loading controls for the Western blots. All experiments were performed in the standard experiment conditions (cells cultured in the above conditions for 3 days in 1% FBS medium after overnight serum starvation). Error bars indicate the standard deviation. *, *p* value < 0.05. The uncropped Western blot figures are presented in Figure S5.

COL11A1 was extracted from A204 (a rhabdomyosarcoma cell line that expresses COL11A1; [21]) scrambled (scrm) control or A204-shCOL11A1 cells, as described previously [9,21]. A204-scrm cells or A204-shCOL11A1 cells were cultured in DMEM supplemented with 10% FBS, 50 µg/mL sodium ascorbate, and 50 µg/mL B-aminopropionitrile (BAPN; A3134-5G, Sigma-Aldrich, St. Louis, MO, USA) for 48 h. Medium, cells, and extracellular matrix proteins on the plate were collected in chilled 0.5 µM acetic acid supplemented with a cocktail of protease inhibitors (2.5 mM EDTA, 0.2 mM PMSF and 10 mM N-ethylmaleimide) and then filtered through a Spetra/Por dialysis membrane (Spectrum labs; San Francisco, CA, USA; #132655) with a pore size of 6–8 kD, resulting in a COL11A1-positive or -negative extract. COL11A1 expression in A204 cells and extract was validated by Western blot (Figure 1A,B). For the cell culture, 5 µg/mL of the extracted COL11A1 or type I collagen (COL1) diluted in PBS (Gibco Life Technologies; Waltham, MA, USA) was used to coat the tissue culture plates for 2 h at 37 °C or overnight at 4 °C. Recipient ovarian cancer cells, which were serum starved overnight prior to the experiments, were trypsinized and plated on collagen-positive and -negative extract coated plates.

To obtain decellularized matrices that are positive and negative for COL11A1, A204-scrm cells or A204-shCOL11A1 were seeded at 30–40% confluency and then stimulated with 50 µg/mL sodium ascorbate supplemented DMEM (with 10% FBS and 1× penicillin/ streptomycin) for 2 days. Then, A204-scrm, A204-shCOL11A1, and regular tissue culture plastic plates were incubated with sterile PBS supplemented with 20 mM EDTA for 10 min at 37 °C. The wash buffer was then aspirated and replaced with sterile PBS supplemented with 30 mM EDTA and 0.5% Triton X-100, which was chosen to de-cellularize the A204-derived matrices due to its effectiveness at de-cellularizing ACL tissue in a previous study [22]. Plates were rocked back and forth and swirled gently in sterile conditions. After 2–3 min, this solution was replaced with fresh sterile PBS supplemented with 30 mM EDTA and 0.5% Triton X-100 and the above steps were repeated. Plates were then washed three times with sterile PBS and then three more times with sterile water. Decellularized matrices were dried in the sterile hood for at least one hour. Expression of COL11A1 in A204 cells and decellularized matrices was validated and quantified by an immunofluorescence plate reader experiment (Figure S1A and Figure 1C–E). Recipient ovarian cancer cells, which were serum starved overnight prior to the experiments, were trypsinized and plated on COL11A1-positive and -negative decellularized matrices.

### 2.3. Human Phospho-Kinase Array

ES2 cells were serum starved overnight and then cultured on COL11A1 positive extract coated plates (at 5 µg/mL) and control (no coat) conditions for 72 h in 1% FBS and 1× penicillin/streptomycin supplemented DMEM. Cells were then collected and lysed in a Human Phospho-Kinase Array kit lysis buffer (R&D Systems, Minneapolis, MN, USA; #ARY003B). A total of 200 µg of protein was then added to the Human Phospho-Kinase Array kit membranes and rocked overnight at 4 °C. Membranes were then incubated with various antibodies and substrates and was as per the kit instructions (R&D Systems, Minneapolis, MN, USA; #ARY003B). The HRP signal from the kinase array membranes were captured with film exposure. 

### 2.4. Immunofluorescence Plate Reader Experiment

Five thousand A204-scrm and A204-shCOL11A1 cells were seeded (per well) in a 96-well plate and cultured in 50 µg/mL sodium ascorbate-supplemented DMEM (with 10% FBS and 1× penicillin/ streptomycin) for 2 days. Half of the wells for each group of cells were then decellularized (see the “Cell Culture on Collagen Extract and Acellular Matrix” method section above). Cells and decellularized groups were then fixed in 10% formalin and stained with COL11A1 antibody (Abcam; Cambridge, MA, USA; #ab64883), DAPI (Invitrogen; CA, USA, # D1306) and a FITC goat anti-mouse conjugated secondary antibody (Gift from Dr. Gang Liu, Albany Medical College, Albany, USA), as per the manufacturer’s recommended immunofluorescence protocol, and then read with a Glomax Explorer Microplate Reader (Promega; Madison, WI, USA). 

### 2.5. Cell Viability Assays

CellTiter-Glo Luminescent cell viability assays (Promega; Madison WI, USA; #G7572) and acid phosphatase (Millipore Sigma; Burlington, MA, USA; #4876) assays were employed to evaluate cell viability using a Glomax Explorer Microplate Reader (Promega; Madison, WI, USA), according to the manufacturer’s protocols. 

### 2.6. Lentiviral shRNA Knockdown

Stable gene knockdown was performed using shRNAs, as described previously [8,9]; the constructs are listed in Table S1. Lipofectamine 2000 (Life technologies; Carlsbad, CA, USA) was used to co-transfect pCMV-ΔR 8.2, pCMV-VSVG, and a lentiviral construct containing shRNA into Lenti-X 293T cells. Then, 24–48 h later, lentivirus-containing media was harvested and filtered through a 0.45 µm PVDF low protein-binding membrane filter (Celltreat; Pepperell, MA, USA). Cancer cells were incubated in the lentivirus-containing medium and with 8 µg/mL polybrene (EMD Millipore; Burlington, MA, USA; #TR-1003-G) for 72 h at 37 °C with 5% CO_2_ followed by 5 µg/mL puromycin (ThermoFisher, Waltham, MA, USA; #A1113803) or hygromycin selection; 100 µg/mL for A2780 cells and 400 µg/mL for ES2 cells (ThermoFisher Waltham, MA, USA; #10687010). Gene knockdown was confirmed by Western blotting and qRT-PCR.

### 2.7. qRT-PCR and Immunoblotting

Total cellular RNA was extracted using the RNeasy mini kit (Qiagen; Hilden, Germany). A total of 1 µg of RNA was reverse transcribed into cDNA using the Quantitect Reverse Transcription Kit (Qiagen; Hilden, Germany). Then, 50 ng of cDNA was amplified using the relevant primers (sequences listed in Table S2) and iQSYBR-Green Supermix (BioRad; Hercules, CA, USA). The qRT-PCR reaction was performed using a CFX96 real-time PCR detection system (BioRad; Hercules, CA, USA) and the data were analyzed by the 2-ΔΔCt formula method.

Total protein was extracted using RIPA cell lysis buffer (Sigma; St. Louis, MO, USA) containing a complete mini protease inhibitor cocktail (Sigma; St. Louis, MO, USA) and PhosSTOP (Sigma; St. Louis, MO, USA). The BCA assay kit (Pierce; Waltham, MA, USA) was used to determine the protein concentration. Samples containing approximately 25 µg of protein were run on 4–20% Mini-PROTEAN® TGX Stain-Free™ Protein Gels (BioRad; Hercules, CA, USA) and transferred onto PVDF membranes followed by blocking and primary and secondary antibody incubations (antibodies used are listed in Table S3). Clarity western ECL substrate (BioRad; Hercules, CA, USA) was added to the membrane and the chemiluminescent signals were detected by a ChemiDoc MP imaging system (BioRad; Hercules, CA, USA) according to the manufacturer’s recommendation.

### 2.8. Xenograft Experiments

All animal experiments were approved by the Albany Medical College’s Institutional Animal Care and Use Committee. Six-week-old female NCr nude mice (Taconic Biosciences; Germantown, NY, USA) were injected intraperitoneally with 1:1 volume of Matrigel and 500,000 A2780CIS-scrambled (scrm) control or A2780CIS-shCOL11A1 cells in serum-free RPMI media. In another experiment, 6-week-old female NCr nude mice (Taconic Biosciences; Germantown, NY, USA) were pre-injected with 100 μL of 0.2 mg/mL COL11A1 extract dissolved in PBS or 100 μL of PBS. Three days post injection, the animals were intraperitoneally injected with a 1:1 volume of Matrigel and 500,000 A2780 cells in serum-free RPMI media. We previously demonstrated that the resultant tumors become resistant to cisplatin by COL11A1 injection using this approach [9]. Similar approaches have been used by others to study the role of collagens in various aspects of tumor progression [23,24,25]. Seven days after tumor cell injection, 3 mg/kg cisplatin was administered to the mice twice every week for 3 weeks by i.p. injection. The control animals were injected with an equivalent amount of vehicle (DMSO). All animals were monitored daily for survival until the experimental endpoint (8 weeks post cancer cells injection) or until death, whichever occurred first. Mice were euthanized by CO_2_ inhalation followed by cervical dislocation. Tumors were collected and processed for histopathological analyses.

### 2.9. Immunohistochemistry

In total, 3 µm formalin-fixed, paraffin-embedded xenograft tumor sections were cut using a microtome (Leica Biosystems, Danvers, MA, USA). Tumor sections were deparaffinized with 3 changes of xylene for 5 min each, followed by tissue hydration using a 100, 95, 70, and 50% graded ethanol series for 3 min each. Sections were rinsed in distilled water twice for 5 min, and then rinsed in high pH sodium citrate buffer (H-3300-250; Vector laboratories, CA, USA) at 95 °C for 20 min. Slides were run under distilled water for 10 min, followed by three 5-min washes using TBST + 0.75% glycine, permeabilized with 0.5% triton X containing wash buffer, washed again, and blocked for 60 min at room temperature using 10% goat serum in the wash buffer. Tissues were incubated overnight at 4 °C with mouse anti-HSP27 (1:50; Cell Signaling-2402; Danvers, MA, USA), rabbit anti-HSP27 p-Ser78 (1:25; Cell Signaling-2405), and rabbit anti-HSP27 p-Ser82 (1:100; Cell Signaling-9709) primary antibodies diluted in wash buffer containing 2% BSA, followed by incubations with goat anti-rabbit or goat anti-mouse secondary Alexafluor 488 antibody (A-11034 and A-11029; Invitrogen; CA, USA) incubation for 1 h at room temperature. After washing, coverslips were mounted using Prolong anti-fade diamond mounting media containing DAPI (P36971; Invitrogen; CA, USA) for nuclear staining. Images were acquired at 4×, 10×, and 20× magnification on an Olympus BX61 upright microscope with a PCO.EDGE 4.2 scientific CMOS camera and analyzed with MetaMorph software (version 7.10.2, Molecular Device).

### 2.10. Patient Data Analysis

Kaplan–Meier curves were generated for ovarian cancer patients expressing high or low expression of the selected genes. HSP27 (HBPB1; 201841_s_at) was inputted into the gene symbol tab and overall survival was computed for serous subtype using the default settings on KM plotter (http://kmplot.com/analysis/index.php?p=service&cancer=ovar). Similarly, to achieve overall survival statistics for the mean expression of HSP27 and COL11A1, COL11A1 (229271_x_at) and HSPB1 (201841_s_at) were inputted into the multiple gene entry tab, followed by “mean expression of the selected probes” option.

### 2.11. Statistical Analysis

Statistical analyses were performed using GraphPad Prism software version 7.0 (GraphPad Software; San Diego, CA, USA). A Student’s T-test was used to compare the means of two groups, and a one-way ANOVA was applied to compare the mean difference of three or more groups. *p* ≤ 0.05 was considered significant.

## 3. Results

### 3.1. Collagen Type XI alpha 1 Induces Heat Shock Protein 27 Phosphorylation and Total Expression in Ovarian Cancer Cells

We previously reported that collagen type XI alpha 1 (COL11A1) confers cisplatin resistance to ovarian cancer cells through activation of Src-Akt-NFkB signaling to upregulate the expression of inhibitor of apoptosis proteins (IAPs) as well as by inducing a metabolic switch that prefers fatty acid metabolism [8,9]. We sought to further identify additional COL11A1 downstream signaling molecules that may mediate COL11A1-induced cisplatin resistance. To do this, we performed a protein kinase array on ES2 cells (an ovarian cancer cell line that expresses low levels of endogenous COL11A1 [8,9]) cultured on COL11A1 extract coated plates and discovered that COL11A1 induced expression of phosphorylated HSP27 at its serine 78 and 82 residues (S78/82) (Figure 1A,B). Even protein loading was confirmed by equivalent expression levels of β-catenin in both groups (Figure S1F). Phosphorylated HSP27 (S78/82) was one of the top upregulated phosphoproteins in ES2 cells cultured on COL11A1, along with phospho-Akt (S473), phospho-Src (Y419), phospho-ERK1/2 (T202/Y204, T185/Y187), and phospho-AMPKα1 (T183) (Table S4), all of which are known downstream effector molecules of COL11A1 [8,9,26]. We then validated these findings through a second kinase array using the same experimental set up and found that phospho-HSP27 (S78/82) was consistently a top upregulated phosphorylated protein in ES2 cells stimulated with COL11A1. Western blot analysis of ovarian cancer cells cultured on collagen extract or decellularized matrices in the presence or absence of COL11A1 demonstrated that COL11A1 increased phosphorylation of both the serine 78 and 82 sites on HSP27 across ES2 and A2780 (a cisplatin-sensitive ovarian cancer cell line that also expresses low endogenous COL11A1 [9]) in decellularized matrix-based experiments (Figure 1C,D) as well as in ES2 and SKOV3 (an ovarian cancer cell line) in COL11A1 extract experiments (Figure S1G,H). COL11A1 also increased the expression of total HSP27 (Figure 1C,D and Figure S1G,H).

In contrast, COL11A1 knockdown (measured by Western blot and RT-PCR (Figure 1E and Figure S1I)) in A2780CIS cells (a cisplatin-resistant ovarian cancer cell line that expresses high levels of endogenous COL11A1 [9]) led to decreased phosphorylated and total HSP27 protein expression (Figure 1E) as well as HSPB1 (a gene encoding HSP27) expression (Figure 1F) compared to the scrambled control cells (as expected). Notably, expression levels of genes encoding other heat shock proteins, such as HSP22, HSPB11, and HSP70, were not altered by COL11A1 knockdown, although COL11A1 depletion did slightly (but significantly) downregulate the expression of a gene encoding HSP60 (Figure 1F). Importantly, type I collagen, the most abundant collagen in the tumor microenvironment, did not increase the total expression nor phosphorylation of HSP27 (at S78/82) in contrast to COL11A1 (Figure 1G), suggesting that an increase of HSP27 expression and phosphorylation might be specific to COL11A1 in ovarian cancer cells. In summary, this data suggests that COL11A1 induces both total and phosphorylated HSP27 in ovarian cancer cells.

### 3.2. COL11A1 Upregulates HSP27 Phosphorylation and Expression through Activation of Discoidin Domain Receptor 2/integrin α1β1-Src-Akt Signaling in Ovarian Cancer

**Figure 2:**
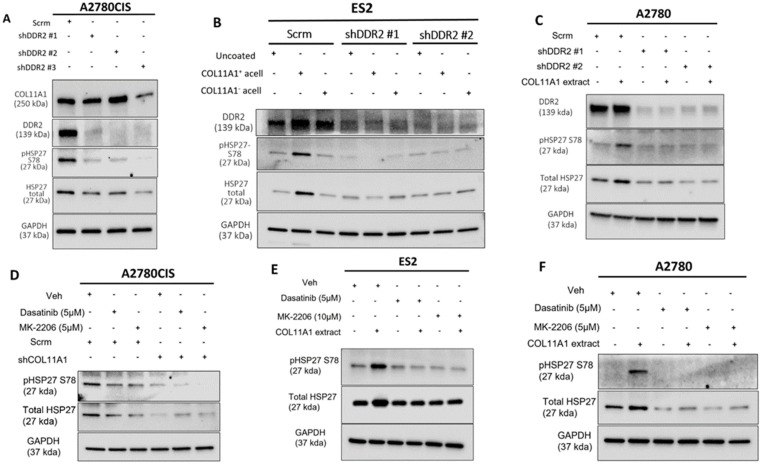
COL11A1 upregulates HSP27 phosphorylation and expression through activation of DDR2/integrin α1β1-Src-Akt signaling in ovarian cancer. (**A**) Western blot of the phosphorylated (at S78) and total HSP27, DDR2, and COL11A1 in A2780CIS-scrambled (scrm) and A2780CIS-shDDR2 cells. (**B**) Western blot of the phosphorylated (at S78) and total HSP27 in ES2-scrm and ES2-shDDR2 #1 and #2 on COL11A1-positive and -negative A204-derived decellularized matrices and control conditions. (**C**) Western blot of the phosphorylated (at S78) and total HSP27 in A2780-scrm and A2780-shDDR2 in the COL11A1-positive extract and control conditions. (**D**) A2780CIS-scrm and A2780CIS-shCOL11A1 cells treated with Dasatinib (5 µM) and MK-2206 (5 µM) for 72 h. (**E**) ES2 cells cultured on the COL11A1-positive extract and control conditions treated with Dasatinib (5 µM) and MK-2206 (10 µM) for 72 h. (**F**) A2780 cells cultured on the COL11A1-positive extract and control conditions treated with Dasatinib (5 µM) and MK-2206 (5 µM) for 72 h. GAPDH was used as a loading control for the Western blots. All experiments were performed in the standard experiment conditions (cells cultured in the above conditions for 3 days in 1% FBS medium after overnight serum starvation). The uncropped Western blot figures are presented in Figure S6.

Our previous studies have shown that COL11A1 confers cisplatin resistance through the collagen cell surface receptors discoidin domain receptor 2 (DDR2)/integrin (ITG) α1β1-Src-Akt signaling [8,9]. Thus, we then sought to determine if COL11A1 engages the same signaling cascade to upregulate total and phosphorylated HSP27. Knockdown of DDR2 was sufficient in attenuating COL11A1 induction of HSP27 expression and phosphorylation in A2780CIS, ES2, and A2780 cells (Figure 2A–C). Knockdown of integrin α1 also attenuated COL11A1 induction of HSP27 in ES2 and A2780 cells (Figure S2A,B). After confirmation that COL11A1 engagement of the DDR2 and ITG-α1β1 collagen receptors induced HSP27 phosphorylation and expression, we then determined what downstream signaling molecules mediate COLL1A1-induced HSP27 expression and phosphorylation. Previously we had determined that activation of the Src and Akt kinases occur following COL11A1-receptor engagement [9]. To investigate whether COL11A1 upregulates total and phosphorylated HSP27 through Src kinase, we inhibited Src activation with a pharmaceutical inhibitor Dasatinib [27]. Dasatinib treatment decreased COL11A1-induced phosphorylation of HSP27 at serine 78 and total expression of HSP27 in A2780CIS cells, ES2 cells, and A2780 cells (Figure 2D–F), Similarly, Akt inhibition through MK2206 treatment also decreased COL11A1-induced phosphorylation of HSP27 at serine 78 and total expression of HSP27 in A2780CIS cells, as well as in ES2 and A2780 cells (Figure 2D–F). These results suggest that COL11A1 upregulates the expression and phosphorylation of HSP27 through DDR2/integrin α1β1 and Src/Akt signaling in ovarian cancer cells.

### 3.3. HSP27 Mediates COL11A1-Induced Cisplatin Resistance in Ovarian Cancer Cells

**Figure 3:**
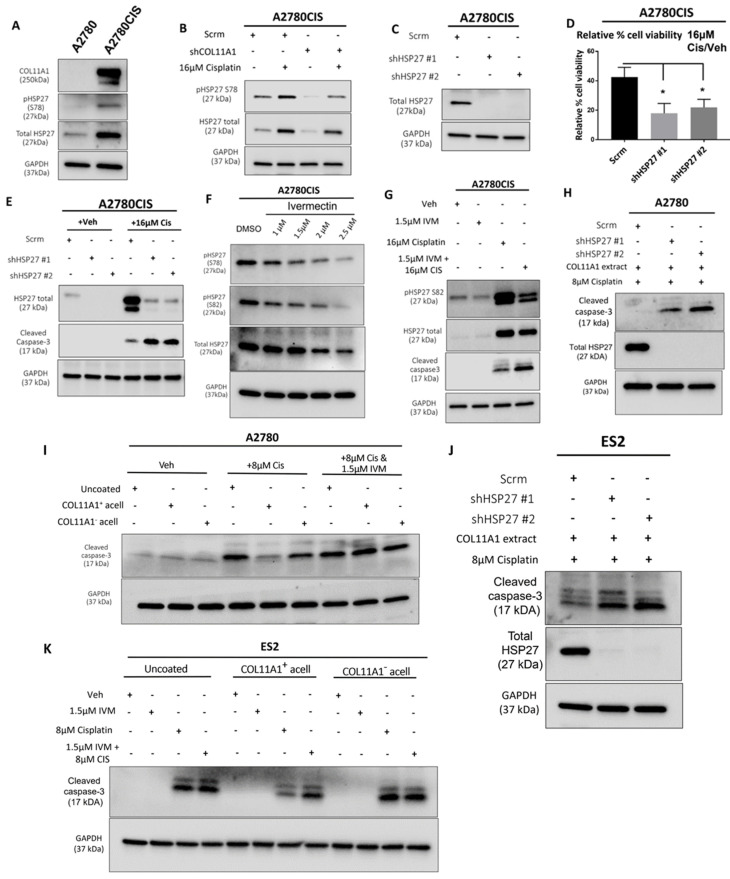
HSP27 mediates COL11A1-induced cisplatin resistance in ovarian cancer cells. (**A**) Western blot of the phosphorylated (at S78) and total HSP27 in A2780 and A2780CIS. (**B**) Western blot of the phosphorylated (at S78) and total HSP27 in A2780CIS-scrm and A2780CIS-shCOL11A1 treated with 16 µM cisplatin. (**C**) Western blot of the total HSP27 in A2780CIS-scrm and A2780CIS-shHSP27 #1 and #2. (**D**) Relative cell viability (measured by Cell titer glo) of A2780CIS-scrm and A2780CIS-shHSP27 #1 and #2 treated with 16 µM cisplatin compared with the respective vehicle-treated control groups (*n* = 3). (**E**) Western blot of total HSP27 and cleaved caspase-3 in A2780CIS-scrm and A2780CIS-shHSP27 #1 and #2 and treated with the vehicle treatment or 16 µM cisplatin. (**F**) Western blot of the phosphorylated (at S78 and S82) and total HSP27 in the vehicle and 1 µM, 1.5 µM, 2 µM, and 2.5 µM ivermectin (IVM)-treated A2780CIS cells. (**G**) Western blot of the phosphorylated (at S82) and total HSP27 and cleaved caspase-3 in the vehicle, 1.5 µM ivermectin (IVM), 16 µM cisplatin, and combination-treated (1.5 µM ivermectin and 16 µM cisplatin) A2780CIS cells. (**H**) Western blot of the total HSP27 and cleaved caspase-3 in A2780-scrm and A2780-shHSP27 #1 and #2 cultured on a COL11A1 extract and treated with 8 µM cisplatin. (**I**) Western blot of and cleaved caspase-3 in A2780 cells cultured on COL11A1-positive and -negative A204-derived decellularized matrices and control conditions treated with 8 µM cisplatin (Cis) and the combination (1.5 µM ivermectin and 8 µM cisplatin (IVM + Cis)) and vehicle treatments. (**J**) Western blot of the total HSP27 and cleaved caspase-3 in ES2-scrm and ES2-shHSP27 #1 and #2 cultured on a COL11A1 extract and treated with 8 µM cisplatin. (**K**) Western blot of cleaved caspase-3 in ES2 cells cultured on COL11A1-positive and -negative A204-derived decellularized matrices and control conditions treated with 1.5 µM ivermectin (IVM), 8 µM cisplatin (Cis), and the combination (1.5 µM ivermectin and 8 µM cisplatin (IVM + Cis)) and vehicle treatments. GAPDH was used as a loading control for the Western blots. All experiments were performed in the standard experiment conditions (cells cultured in the above conditions for 3 days in 1% FBS medium after overnight serum starvation). Error bars indicate the standard deviation. *, *p* value < 0.05. The uncropped Western blot figures are presented in Figure S7.

HSP27 is associated with cisplatin resistance in ovarian cancer [19,28]. Consistent with this, we observed higher levels of total and phosphorylated HSP27 in the A2780CIS cisplatin-resistant cell line compared to A2780, the cisplatin-sensitive parental cell line (Figure 3A). Since drugs and other forms of cytotoxic stress have been reported to induce heat shock proteins [29,30], which are known to play a role in the cellular stress response [31], we also checked whether the levels of total and phosphorylated HSP27 are changed upon cisplatin treatment. Treatment of the A2780CIS scramble control (scrm) and A2780CIS-shCOL11A1 cells with cisplatin increases total and phosphorylated HSP27, with the A2780CIS-scrm cells maintaining higher levels of total and phosphorylated HSP27 compared to A2780CIS-shCOL11A1 cells (Figure 3B). We next investigated if HSP27 mediates COL11A1-induced cisplatin resistance in ovarian cancer cells. HSP27 was first stably depleted by lentiviral transduction in A2780CIS cells (Figure 3C). HSP27 knockdown A2780CIS cells were significantly more sensitive to cisplatin treatment relative to the scrambled control cells, as evident by a decreased cell viability and increased cleaved caspase-3 levels in knockdown groups relative to the scrambled control cells post cisplatin treatment (Figure 3D,E). To give our results clinical relevance, we employed the use of ivermectin, an FDA-approved antiparasitic drug [32], which potently inhibits HSP27 phosphorylation [33]. Indeed, ivermectin treatment inhibited HSP27 phosphorylation in A2780CIS cells (Figure 3F) at both S78 and 82 residues at 1.5 µM, which we also found to be the approximate IC50 drug concentration for ivermectin in A2780CIS cells (Figure S3A). We observed that HSP27 inhibition with ivermectin sensitized A2780CIS to cisplatin treatment, indicated by increased levels of cleaved caspase-3 (Figure 3G). HSP27 knockdown or inhibition with ivermectin also sensitized A2780 and ES2 cells cultured on COL11A1 to cisplatin treatment (Figure 3H–K). Collectively, these results suggest that HSP27 mediates COL11A1-induced cisplatin resistance in ovarian cancer cells.

### 3.4. HSP27 Regulates NFkB Activity and Inhibitors of Apoptosis Expression

**Figure 4:**
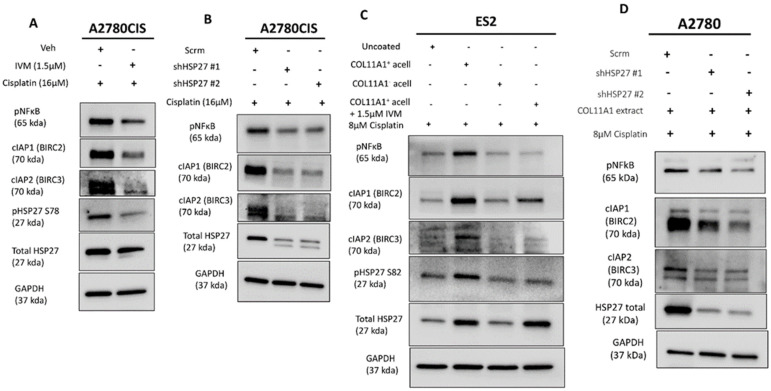
HSP27 regulates NFκB activity and IAP expression. (**A**) Western blot of the phosphorylated (active) NFκB, cIAP1 (BIRC2), and cIAP2 (BIRC3) in cisplatin-stimulated A2780CIS cells treated either with the vehicle (Veh) or 1.5 µM ivermectin (IVM) treatments. (**B**) Western blot of the phosphorylated (active) NFκB, cIAP1 (BIRC2), and cIAP2 (BIRC3) in cisplatin-treated A2780CIS-scrm and A2780CIS-shHSP27 #1 and #2 cells. (**C**) Western blot of phosphorylated (active) NFκB, cIAP1 (BIRC2), and cIAP2 (BIRC3) in cisplatin-treated ES2 cells cultured in the control, COL11A1-positive a-cellular matrix, COL11A1-negative a-cellular matrix, and COL11A1-positive a-cellular matrix plus the 1.5 µM ivermectin (IVM)-treated conditions. (**D**) Western blot of phosphorylated (active) NFκB, cIAP1 (BIRC2), and cIAP2 (BIRC3) in cisplatin-treated A2780-scrm and A2780-shHSP27 #1 and #2 cells cultured on COL11A1. GAPDH was used as a loading control for the Western blots. All experiments were performed in the standard experiment conditions (cells cultured in the above conditions for 3 days in 1% FBS medium after overnight serum starvation). The uncropped Western blot figures are presented in Figure S8.

We then determined how HSP27 might mediate COL11A1-induced cisplatin resistance. We previously established that COL11A1 promotes cisplatin resistance in ovarian cancer cells through activation of NFkB and upregulation of specific inhibitors of apoptosis (IAPs; BIRC2, BIRC3, and XIAP) [9]. Other research groups have reported that HSP27 promotes NFkB activity as well as IAP activity (albeit through an indirect way via Smac inhibition) in multiple cell types [34,35,36]. Thus, it was important to determine whether HSP27 also positively regulates NFkB activity and IAP expression in ovarian cancer cells. HSP27 inhibition through ivermectin treatment or HSP27 knockdown decreased the NFkB activity (as indicated by a decrease in phosphorylated NFkB at its serine 536 residue) and IAP (BIRC2 and BIRC3) expression in cisplatin-treated A2780CIS cells (Figure 4A,B) as well as in cisplatin-treated ES2 and A2780 cells cultured on COL11A1 (Figure 4C,D). These results suggest that HSP27 might mediate COL11A1-induced cisplatin resistance through activation of NFkB and upregulation of IAPs (BIRC2 and BIRC3) in ovarian cancer cells.

### 3.5. HSP27 Expression and Phosphorylation Is Increased by COL11A1 in Xenograft Tumors and Is a Marker for Poor Prognosis

**Figure 5:**
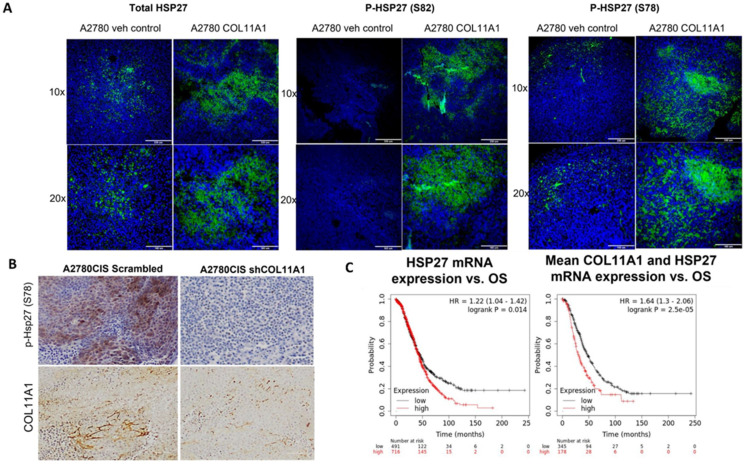
HSP27 expression and phosphorylation is upregulated in vivo in tumor xenografts and in ovarian cancer patients. (**A**) Representative immunofluorescence images of total and p-HSP27 (S78 and 82) in xenograft tumors generated from A2780/vehicle-injected mice and A2780/COL11A1-injected mice. Green: HSP27 (total and phospho); blue: DAPI. Scale bars: 330 µm (10×) and 165 µm (20×). (**B**) Representative images of IHC staining of p-HSP27 (S78) and COL11A1 in A2780CIS-scrm and A2780-shCOL11A1 tumors. Scale bar: 100 µm. (**C**) Overall survival in patients expressing high levels of HSP27 mRNA compared to the low-expressing group (left). Overall survival in patients expressing high mean expression of COL11A1 and HSP27 mRNAs compared to the low-expressing group (right).

To determine whether HSP27 expression and phosphorylation is upregulated by COL11A1 in vivo, we intraperitoneally injected nude mice with PBS or COL11A1 extract followed by injection of A2780 human ovarian cancer cells, which were then later challenged with cisplatin treatment. When mice developed tumors, they were euthanized, and tumors were isolated. Paraffin-embedded tumor sections were stained for total HSP27 and phospho-HSP27 (Ser 78 and 82) and analyzed by immunofluorescence (Figure 5A). In a separate experiment, we generated A2780CIS-scrm and A2780CIS-shCOL11A1 xenograft tumors and performed IHC on them for phospho-HSP27 (Ser 78) (Figure 5B). In both xenograft tumors, the presence of COL11A1 predominantly increased HSP27 phosphorylation (Figure 5A,B), in concordance with our in vitro data.

**Figure 6:**
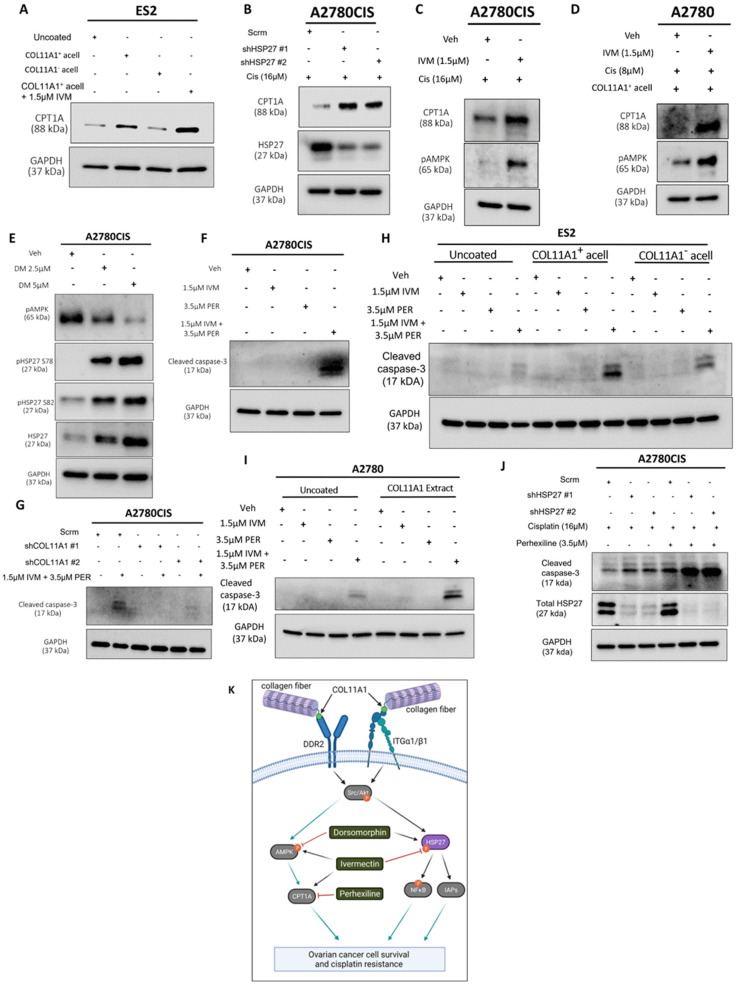
HSP27 synergizes with FAO to enhance cisplatin resistance. (**A**) Western blot of CPT1A in ES2 cells cultured in the control, COL11A1-positive a-cellular matrix, COL11A1-negative a-cellular matrix, and COL11A1-positive a-cellular matrix plus the 1.5 µM ivermectin (IVM)-treated conditions. (**B**) Western blot of CPT1A and HSP27 in cisplatin-treated A2780CIS-scrm and A2780CIS-shHSP27 #1 and #2 cells. (**C**) Western blot of active (phosphorylated) AMPK and CPT1A in the A2780CIS vehicle (Veh) and 1.5 µM ivermectin (IVM)-treated cells treated with 16 µM cisplatin (Cis). (**D**) Western blot of active (phosphorylated at T172) AMPK and CPT1A in the A2780 vehicle and 1.5 µM ivermectin (IVM)-treated cells treated with 8 µM cisplatin cultured on COL11A1-positive a-cellular matrices (Cis). (**E**) Western blot of active (phosphorylated) AMPK and phosphorylated (at S78 and S82) and total HSP27 in the A2780CIS vehicle (Veh) and 2.5 µM and 5 µM dorsomorphin (DM)-treated cells. (**F**) Western blot of cleaved caspase-3 in A2780CIS cells treated with the vehicle (Veh), 1.5 µM ivermectin (IVM), 3.5 µM perhexiline (PER), and combination treatment of 1.5 µM ivermectin and 3.5 µM perhexiline (IVM + PER). (**G**) Western blot of cleaved caspase-3 in A2780CIS-scrm and A2780CIS-shCOL11A1 #1 and #2 cells treated with the vehicle (Veh) and combination treatments of 1.5 µM ivermectin and 3.5 µM perhexiline (IVM + PER). (**H**) Western blot of cleaved caspase-3 in ES2 cells treated with the vehicle (Veh), 1.5 µM ivermectin (IVM), 3.5 µM perhexiline (PER), and combination treatment of 1.5 µM ivermectin and 3.5 µM perhexiline (IVM + PER) cultured in the control, COL11A1-positive a-cellular matrix, and COL11A1-negative a-cellular matrix conditions. (**I**) Western blot of cleaved caspase-3 in A2780 cells treated with the vehicle (Veh), 1.5 µM Ivermectin, 3.5 µM perhexiline, and combination treatment of 1.5 µM Ivermectin and 3.5 µM perhexiline cultured in the control and COL11A1-positive extract conditions. (**J**) Western blot of cleaved caspase-3 and HSP27 in A2780CIS-scrm and A2780CIS-shHSP27 #1 and #2 cells treated with 16 µM cisplatin, and combination 16 µM cisplatin plus 3.5 µM perhexiline treatments. (**K**) A proposed mechanism by which COL11A1 upregulates HSP27 expression and phosphorylation, as well as inhibitors of the COL11A1 signaling pathway plus their target molecules. Black arrows indicate the proposed signaling sequences; grey arrows indicate the signaling cascades based on the literature; and red flat head arrows indicate drug inhibition. GAPDH was used as a loading control for the Western blots. All experiments were performed in the standard experiment conditions (cells cultured in the above conditions for 3 days in 1% FBS medium after overnight serum starvation). The uncropped Western blot figures are presented in Figure S9.

To determine whether high levels of HSP27 expression is associated with poor prognosis, we correlated the HSP27 mRNA levels with overall survival in 1207 high-grade serous ovarian cancer patients using the Kaplan–Meier plotter (https://kmplot.com) and discovered that high HSP27 expression is associated with poor overall survival in high-grade serous ovarian cancer patients (HR + 1.22 (1.04–1.42), logrank *p* = 0.014; Figure 5C). Mean expression of COL11A1 and HSP27 mRNA was also associated with poor overall survival in high-grade serous ovarian cancer patients (HR = 1.64 (1.3–2.06), logrank *p* = 2.5 × 10^5^; Figure 6C). Collectively, these results suggest that HSP27, whose expression and activity is upregulated by COL11A1, is a marker for poor prognosis in ovarian cancer. 

### 3.6. HSP27 Synergizes with Fatty Acid Oxidation to Enhance Cisplatin Resistance

A recent study showed that fatty acid oxidation (FAO) compensates for inhibition (with the drug AUY922) of another heat shock protein, HSP90, in prostate cancer cells [37]. We previously reported that FAO is upregulated by COL11A1 to confer cisplatin resistance in ovarian cancer cells [8]. After identifying HSP27 as a novel downstream molecule of COL11A1 that promotes cisplatin resistance, it was necessary to test whether HSP27 also regulates FAO in ovarian cancer cells. Consistent with our previous report [8], ES2 cells cultured on COL11A1 showed increased expression of CPT1A (Figure 6A), the rate-limiting enzyme of FAO [38]. Interestingly, when these cells were treated with the HSP27 inhibitor ivermectin, they further increased CPT1A expression, indicative of increased FAO (Figure 6A). HSP27 knockdown also upregulated FAO in cisplatin-treated A2780CIS cells (Figure 6B). To test the hypothesis that ovarian cancer cells might upregulate FAO in response to HSP27 inhibition, similar to prostate cancer cells that have been shown to upregulate FAO in response to HSP90 inhibition [37], co-treatment of ivermectin and cisplatin increased the levels of phosphorylated AMP kinase (at its T172 residue), a master regulator of FAO [39], compared to the cisplatin-only treated controls in A2780CIS cells as well as in A2780 and ES2 cells cultured on COL11A1 (Figure 6C,D and Figure S4A). We also observed that co-treatment of ivermectin and cisplatin increased CPT1A expression in A2780CIS cells as well as in A2780 cells cultured on COL11A1 (Figure 6C,D). These results suggest a potential mechanism by which HSP27 inhibition could lead to FAO upregulation. In addition to these results, a dose-dependent increase in HSP27 (total and phosphorylated) was evident in A2780CIS cells treated with increasing concentrations of the AMPK inhibitor dorsomorphin (Figure 6E), suggesting that AMPK and HSP27 may be activated in divergent COL11A1 signaling pathways and/or may compensate for each other if one is inhibited.

Next, we tested the effect of dual inhibition of FAO and HSP27 in ovarian cancer cell viability. Interestingly, we observed that the HSP27 inhibitor ivermectin synergized with an FAO inhibitor perhexiline ([40]; a CPT1 inhibitor) in killing A2780CIS cells (Figure 6F,G and Figure S4B,C). We also observed a similar synergy between ivermectin and perhexiline in killing ES2 and A2780 cells cultured on COL11A1 (Figure 6H,I). Furthermore, A2780CIS-shHSP27 cells were more sensitive to perhexiline and cisplatin combination treatment compared to A2780CIS-scrm cells (Figure 6J). These results suggest that ovarian cancer cells cultured on COL11A1 rely more on FAO if HSP27 is inhibited, providing a potential combination therapy for cisplatin-resistant ovarian cancer.

## 4. Discussion

It has become increasingly clear that dysregulated ECM contributes to cancer progression and chemotherapy resistance [15]. Collagen type IX alpha 1 (COL11A1) has emerged as not only a biomarker associated with poor clinical outcomes in ovarian cancer, but also an important molecular player that promotes ovarian cancer cisplatin resistance [8,9,10]. COL11A1 is mainly expressed by cancer-associated fibroblasts adjacent to cancer cells in ovarian cancer [7], although there is also evidence that cisplatin-resistant ovarian cancer cells also express high levels of endogenous COL11A1 [8,9,10]. COL11A1 is subunit of the collagen type XI protein, which is a nucleator of collagen fibers [5]. In its natural state, only the N-terminal domain of COL11A1 is exposed [41], which may be the only accessible part of COL11A1 and thus the only domain that can bind and activate cell surface receptors. However, how COL11A1 is incorporated into the extracellular matrix and induces cellular signaling in a cancer context is still unknown. Although there have been efforts to detail out the mechanisms by which COL11A1 confers cisplatin resistance to ovarian cancer cells [8,9,10], there are still knowledge gaps in the COL11A1 signaling pathway. This study identifies the small heat shock protein 27 (HSP27) as a novel COL11A1 effector molecule that mediates COL11A1-induced cisplatin resistance in ovarian cancer cells. We demonstrated that COL11A1 upregulates total and phosphorylated HSP27 through activation of DDR2/α1β1 integrin-Src-Akt signaling (Figure 6K). We also discovered that HSP27 can mediate COL11A1-induced cisplatin resistance through activation of NFκB and upregulation of IAPs. Additionally, we observed that HSP27 inhibition synergizes with FAO inhibition in killing cisplatin-resistant ovarian cancer cells. These results might yield novel effective therapies for COL11A1-high cancers, which are associated with poor survival, recurrence, and chemoresistance [8,9,10].

Few studies have focused on the relationship between the ECM and HSP27. Here we showed that COL11A1, and not type I collagen, upregulates total and phosphorylated HSP27 in ovarian cancer cells. Other research groups have shown that type I collagen can induce HSP27 phosphorylation in platelets [42,43] and the W1 ovarian cancer cell line [44]. Differences in these studies and our results could be explained by receptor expression in these cells. For example, von Rekowski et al. [44] demonstrated that type I collagen induces HSP27 phosphorylation through DDR1. In addition to type I collagen, other studies have shown that another ECM protein, SPARC, also stimulates HSP27 phosphorylation [45,46]. 

Understanding which cancers may likely benefit from HSP27 inhibition therapy by proxy of the relevant biomarkers (such as COL11A1, SPARC, and potentially type I collagen) may lead to more treatment successes.

Our study also showed that the collagen receptors DDR2 and integrin α1β1 mediate COL11A1 induction of total and phosphorylated HSP27. We further demonstrated that Src and Akt kinases also mediate expression and phosphorylation of COL11A1-induced HSP27. It is known that the MAP kinases MK2, MK3, and MK5 phosphorylate HSP27 at its serine 78 and 82 residues, as do PKA, Akt, PKC, and PKD [47]. Indeed, we showed that the Akt inhibitor MK2206 can attenuate COL11A1-induced HSP27 phosphorylation, suggesting that Akt induces phosphorylation of HSP27 in this setting. However, it remains to be determined how exactly COL11A1 induces phosphorylation and total expression of HSP27.

Our results show that HSP27 mediates COL11A1 activation of NFkB and increased expression of IAPs. HSP27 aids in the activation of NFkB by enhancing the degradation of IkBα, a regulatory protein of NFkB, and also increases the activity of IAPs by inhibiting the release of SMAC/Diablo proteins from the mitochondria [34,35]. It is tempting to speculate that a possible mechanism by which ovarian cancer cells stimulated by COL11A1 maintain resistance to cisplatin involves upregulation of total and phosphorylated HSP27, which in turn activates NFkB and subsequentially leads to an increase in IAP expression. We also showed that HSP27 expression is associated with cisplatin resistance. HSP27 is upregulated by cisplatin treatment and confers cisplatin resistance in ovarian cancer cells. How exactly cisplatin treatment upregulates HSP27 remains to be determined. Although the link between HSP27 and cancer cell chemoresistance has been clearly established [11,12,13,19,20], our study demonstrates HSP27 is a novel mediator of COL11A1-driven cisplatin resistance and provides a potential mechanism of how HSP27 becomes upregulated in ovarian cancer.

## 5. Conclusions

In summary, we demonstrated that HSP27 is a novel COL11A1 downstream effector molecule that may be inhibited to treat COL11A1-positive cisplatin-resistant ovarian cancers. We determined that DDR2/integrin α1β1 and Src/Akt signaling mediate COL11A1 induction of total and phosphorylated HSP27, and that HSP27 mediates COL11A1-induced ovarian cancer cisplatin resistance. We also show that HSP27 mediates COL11A1 induction of NFkB and IAPs, which have been shown by our group in a previous publication to mediate COL11A1-stimulated cisplatin resistance. Finally, we present evidence that FAO may be a compensatory mechanism to HSP27 inhibition. Overall, this study identifies HSP27 as a potential target to treat cisplatin-resistant ovarian cancer patients who often overexpress COL11A1.

## Data Availability

Data is contained within the article and supplementary material.

## Supplementary Materials

The following are available online at https://www.mdpi.com/article/10.3390/cancers13194855/s1, Figure S1: HSP27 as a novel downstream molecule of COL11A1, Figure S2: DDR2/ITGA1 and Src/Akt mediate COL11A1-induced HSP27, Figure S3: HSP27 synergizes with FAO to enhance cisplatin resistance, Figure S4: HSP27 synergizes with FAO to enhance cisplatin resistance, Figure S5–S9: The uncropped western blot figures of Figure 1, Figure 2, Figure 3, Figure 4 and Figure 6, Figure S10–S12: The uncropped western blot figures of Figures S1–S3, Table S1: Lentiviral constructs used in this study, Table S2: Real-time PCR primer sequences used in this study, Table S3: List of antibodies and reagents used in this study, Table S4: Top ten differentially regulated proteins from Protein Kinase array.

## References

[1] Cho A., Howell V.M., Colvin E.K. (2015). The extracellular matrix in epithelial ovarian cancer—A piece of a puzzle. Front. Oncol..

[2] Januchowski R., Swierczewska M., Sterzynska K., Wojtowicz K., Nowicki M., Zabel M. (2016). Increased Expression of Several Collagen Genes is Associated with Drug Resistance in Ovarian Cancer Cell Lines. J. Cancer.

[3] Sherman-Baust C.A., Weeraratna A.T., Rangel L.B.A., Pizer E.S., Cho K.R., Schwartz D.R., Shock T., Morin P.J. (2003). Remodeling of the extracellular matrix through overexpression of collagen VI contributes to cisplatin resistance in ovarian cancer cells. Cancer Cell.

[4] Zhang J.G., Zhang J.H., Wang F.C., Xu X.L., Li X., Guan W.C., Men T., Xu G.X. (2021). Overexpressed COL5A1 is correlated with tumor progression, paclitaxel resistance, and tumor-infiltrating immune cells in ovarian cancer. J. Cell. Physiol..

[5] Fernandes R.J., Weis M., Scott M.A., Seegmiller R.E., Eyre D.R. (2007). Collagen XI chain misassembly in cartilage of the chondrodysplasia (cho) mouse. Matrix Biol..

[6] Wenstrup R.J., Smith S.M., Florer J.B., Zhang G.Y., Beason D.P., Seegmiller R.E., Soslowsky L.J., Birk D.E. (2011). Regulation of Collagen Fibril Nucleation and Initial Fibril Assembly Involves Coordinate Interactions with Collagens V and XI in Developing Tendon. J. Biol. Chem..

[7] Vazquez-Villa F., Garcia-Ocana M., Galvan J.A., Garcia-Martinez J., Garcia-Pravia C., Menedez-Rodriguez P., Gonzalez-del Rey C., Barneo-Serra L., de los Toyos J.R. (2015). COL11A1/(pro)collagen 11A1 expression is a remarkable biomarker of human invasive carcinoma-associated stromal cells and carcinoma progression. Tumor Biol..

[8] Nallanthighal S., Rada M., Heiserman J.P., Cha J.N., Sage J., Zhou B., Yang W., Hu Y., Korgaonkar C., Hanos C.T. (2020). Inhibition of collagen XI alpha 1-induced fatty acid oxidation triggers apoptotic cell death in cisplatin-resistant ovarian cancer. Cell Death Dis..

[9] Rada M., Nallanthighal S., Cha J., Ryan K., Sage J., Eldred C., Ullo M., Orsulic S., Cheon D.J. (2018). Inhibitor of apoptosis proteins (IAPs) mediate collagen type XI alpha 1-driven cisplatin resistance in ovarian cancer. Oncogene.

[10] Wu Y.H., Chang T.H., Huang Y.F., Huang H.D., Chou C.Y. (2014). COL11A1 promotes tumor progression and predicts poor clinical outcome in ovarian cancer. Oncogene.

[11] Baylot V., Andrieu C., Katsogiannou M., Taieb D., Garcia S., Giusiano S., Acunzo J., Iovanna J., Gleave M., Garrido C. (2011). OGX-427 inhibits tumor progression and enhances gemcitabine chemotherapy in pancreatic cancer. Cell Death Dis..

[12] Liu C.L., Chen S.F., Wu M.Z., Jao S.W., Lin Y.S., Yang C.Y., Lee T.Y., Wen L.W., Lan G.L., Nieh S. (2016). The molecular and clinical verification of therapeutic resistance via the p38 MAPK-Hsp27 axis in lung cancer. Oncotarget.

[13] Soleimani A., Jalili-Nik M., Avan A., Ferns G.A., Khazaei M., Hassanian S.M. (2019). The role of HSP27 in the development of drug resistance of gastrointestinal malignancies: Current status and perspectives. J. Cell. Physiol..

[14] Bodzek P., Damasiewicz-Bodzek A., Janosz I., Witek L., Olejek A. (2021). Heat shock protein 27 (hsp27) in patients with ovarian cancer. Ginekol. Pol..

[15] Konsgen D., Klinkmann G., Kaul A., Diesing K., Sehouli J., Braicu I., Sumnig A., Erb H.H.H., Stope M.B., Mustea A. (2020). Soluble heat-shock protein 27 in blood serum is a non-invasive prognostic biomarker for ovarian cancer. Eur. J. Obstet. Gyn. Reprod. B.

[16] Langdon S.P., Rabiasz G.J., Hirst G.L., King R.J.B., Hawkins R.A., Smyth J.F., Miller W.R. (1995). Expression of the heat shock protein HSP27 in human ovarian cancer. Clin. Cancer Res..

[17] Yang T., Xu P.F., Gu L.Z., Xu Z.Y., Ge W.H., Li Q., Xu F.F. (2019). Quantitative assessment of serum heat shock protein 27 for the diagnosis of epithelial ovarian cancer using targeted proteomics coupled with immunoaffinity enrichment. Clin. Chim. Acta.

[18] Zhao M., Shen F., Yin Y.X., Yang Y.Y., Xiang D.J., Chen Q. (2012). Increased Expression of Heat Shock Protein 27 Correlates with Peritoneal Metastasis in Epithelial Ovarian Cancer. Reprod. Sci..

[19] Lu H., Sun C.Y., Zhou T., Zhou B., Guo E.S., Shan W.Y., Xia M., Li K.Z., Weng D.H., Meng L. (2016). HSP27 Knockdown Increases Cytoplasmic p21 and Cisplatin Sensitivity in Ovarian Carcinoma Cells. Oncol. Res..

[20] Song T.F., Zhang Z.F., Liu L., Yang T., Jiang I., Li P.L. (2009). Small Interfering RNA-mediated Silencing of Heat Shock Protein 27 (HSP27) Increases Chemosensitivity to Paclitaxel by Increasing Production of Reactive Oxygen Species in Human Ovarian Cancer Cells (HO8910). J. Int. Med. Res..

[21] Kleman J.P., Hartmann D.J., Ramirez F., Vanderrest M. (1992). The Human Rhabdomyosarcoma Cell-Line A204 Lays down a Highly Insoluble Matrix Composed Mainly of Alpha-1 Type-Xi and Alpha-2 Type-V Collagen Chains. Eur. J. Biochem..

[22] Vavken P., Joshi S., Murray M.M. (2009). TRITON-X Is Most Effective among Three Decellularization Agents for ACL Tissue Engineering. J. Orthop. Res..

[23] Jia Y., Liu M., Huang W., Wang Z., He Y., Wu J., Ren S., Ju Y., Geng R., Li Z. (2012). Recombinant human endostatin endostar inhibits tumor growth and metastasis in a mouse xenograft model of colon cancer. Pathol. Oncol. Res..

[24] Levental K.R., Yu H., Kass L., Lakins J.N., Egeblad M., Erler J.T., Fong S.F., Csiszar K., Giaccia A., Weninger W. (2009). Matrix crosslinking forces tumor progression by enhancing integrin signaling. Cell.

[25] Roth J.M., Akalu A., Zelmanovich A., Policarpio D., Ng B., MacDonald S., Formenti S., Liebes L., Brooks P.C. (2005). Recombinant alpha2(IV)NC1 domain inhibits tumor cell-extracellular matrix interactions, induces cellular senescence, and inhibits tumor growth in vivo. Am. J. Pathol..

[26] Zhang B.H., Zhang C., Yang X.T., Chen Y., Zhang H.Q., Liu J.S., Wu Q.C. (2018). Cytoplasmic collagen XII as a prognostic biomarker in esophageal squamous cell carcinoma. Cancer Biol. Ther..

[27] Schittenhelm M.M., Shiraga S., Schroeder A., Corbin A.S., Griffith D., Lee F.Y., Bokemeyer C., Deininger M.W.N., Druker B.J., Heinrich M.C. (2006). Dasatinib (BMS-354825), a dual SRC/ABL kinase inhibitor, inhibits the kinase activity of wild-type, juxtamembrane, and activation loop mutant KIT Isoforms associated with human malignancies. Cancer Res..

[28] Yamamoto K., Okamoto A., Isonishi S., Ochiai K., Ohtake Y. (2001). Heat shock protein 27 was up-regulated in cisplatin resistant human ovarian tumor cell line and associated with the cisplatin resistance. Cancer Lett..

[29] Lee Y.J., Dewey W.C. (1988). Thermotolerance induced by heat, sodium arsenite, or puromycin: Its inhibition and differences between 43 degrees C and 45 degrees C. J. Cell. Physiol..

[30] Vargas-Roig L.M., Gago F.E., Tello O., Aznar J.C., Ciocca D.R. (1998). Heat shock protein expression and drug resistance in breast cancer patients treated with induction chemotherapy. Int. J. Cancer.

[31] Samali A., Orrenius S. (1998). Heat shock proteins: Regulators of stress response and apoptosis. Cell Stress Chaperones.

[32] Laing R., Gillan V., Devaney E. (2017). Ivermectin—Old Drug, New Tricks?. Trends Parasitol..

[33] Nappi L., Aguda A.H., Nakouzi N.A., Lelj-Garolla B., Beraldi E., Lallous N., Thi M., Moore S., Fazli L., Battsogt D. (2020). Ivermectin inhibits HSP27 and potentiates efficacy of oncogene targeting in tumor models. J. Clin. Investig..

[34] Chauhan D., Li G.L., Hideshima T., Podar K., Mitsiades C., Mitsiades N., Catley L., Tai Y.T., Hayashi T., Shringarpure R. (2003). Hsp27 inhibits release of mitochondrial protein Smac in multiple myeloma cells and confers dexamethasone resistance. Blood.

[35] Parcellier A., Schmitt E., Gurbuxani S., Seigneurin-Berny D., Pance A., Chantome A., Plenchette S., Khochbin S., Solary E., Garrido C. (2003). HSP27 is a ubiquitin-binding protein involved in I-kappa B alpha proteasomal degradation. Mol. Cell. Biol..

[36] Wei L., Liu T.T., Wang H.H., Hong H.M., Yu A.L., Feng H.P., Chang W.W. (2011). Hsp27 participates in the maintenance of breast cancer stem cells through regulation of epithelial-mesenchymal transition and nuclear factor-kappa B. Breast Cancer Res..

[37] Nassar Z.D., Mah C.Y., Centenera M.M., Irani S., Sadowski M.C., Scott J.S., Nguyen E.V., Nagarajan S.R., Moldovan M., Lynn D.J. (2020). Fatty Acid Oxidation Is an Adaptive Survival Pathway Induced in Prostate Tumors by HSP90 Inhibition. Mol. Cancer Res..

[38] Zammit V.A. (2008). Carnitine palmitoyltransferase 1: Central to cell function. IUBMB Life.

[39] Jeon S.M. (2016). Regulation and function of AMPK in physiology and diseases. Exp. Mol. Med..

[40] Ashrafian H., Horowitz J.D., Frenneaux M.P. (2007). Perhexiline. Cardiovasc. Drug. Rev..

[41] Brown R.J., Mallory C., McDougal O.M., Oxford J.T. (2011). Proteomic analysis of Col11a1-associated protein complexes. Proteomics.

[42] Kageyama Y., Doi T., Akamatsu S., Kuroyanagi G., Kondo A., Mizutani J., Otsuka T., Tokuda H., Kozawa O., Ogura S. (2013). Rac regulates collagen-induced HSP27 phosphorylation via p44/p42 MAP kinase in human platelets. Int. J. Mol. Med..

[43] Tokuda H., Kuroyanagi G., Tsujimoto M., Enomoto Y., Matsushima-Nishiwaki R., Onuma T., Kojima A., Doi T., Tanabe K., Akamatsu S. (2015). Release of Phosphorylated HSP27 (HSPB1) from Platelets Is Accompanied with the Acceleration of Aggregation in Diabetic Patients. PLoS ONE.

[44] von Rekowski K.W., Konig P., Henze S., Schlesinger M., Zawierucha P., Januchowski R., Bendas G. (2020). Insight into Cisplatin-Resistance Signaling of W1 Ovarian Cancer Cells Emerges mTOR and HSP27 as Targets for Sensitization Strategies. Int. J. Mol. Sci..

[45] Golembieski W.A., Thomas S.L., Schultz C.R., Yunker C.K., Mcclung H.M., Lemke N., Cazacu S., Barker T., Sage E.H., Brodie C. (2008). HSP27 mediates SPARC-induced changes in glioma morphology, migration, and invasion. Glia.

[46] Schultz C.R., Golembieski W.A., King D.A., Brown S.L., Brodie C., Rempel S.A. (2012). Inhibition of HSP27 alone or in combination with pAKT inhibition as therapeutic approaches to target SPARC-induced glioma cell survival. Mol. Cancer.

[47] Katsogiannou M., Andrieu C., Rocchi P. (2014). Heat shock protein 27 phosphorylation state is associated with cancer progression. Front. Genet..

