# Supplementary material for: Heat Shock Protein 27, a Novel Downstream Target of Collagen Type XI alpha 1, Synergizes with Fatty Acid Oxidation to Confer Cisplatin Resistance in Ovarian Cancer Cells
Source: Cancers (Basel). 2021 Sep 28;13(19):4855. doi: 10.3390/cancers13194855 (PMC8508313; doi:10.3390/cancers13194855)
Supplement: Supplementary file 1 [file cancers-13-04855-s001.zip › cancers-1372062-supplementary.pdf]

Supplementary Materials: Heat Shock Protein 27, a Novel Downstream Target of Collagen Type XI alpha 1, Synergizes with Fatty Acid Oxidation to Confer Cisplatin Resistance in Ovarian Cancer Cells

James Patrick Heiserman, Sameera Nallanthighal, Cody C. Gifford, Kayla Graham, Rohan Samarakoon, Chao Gao, Jessica J. Sage, Wenzheng Zhang, Paul J. Higgins and Dong-Joo Cheon

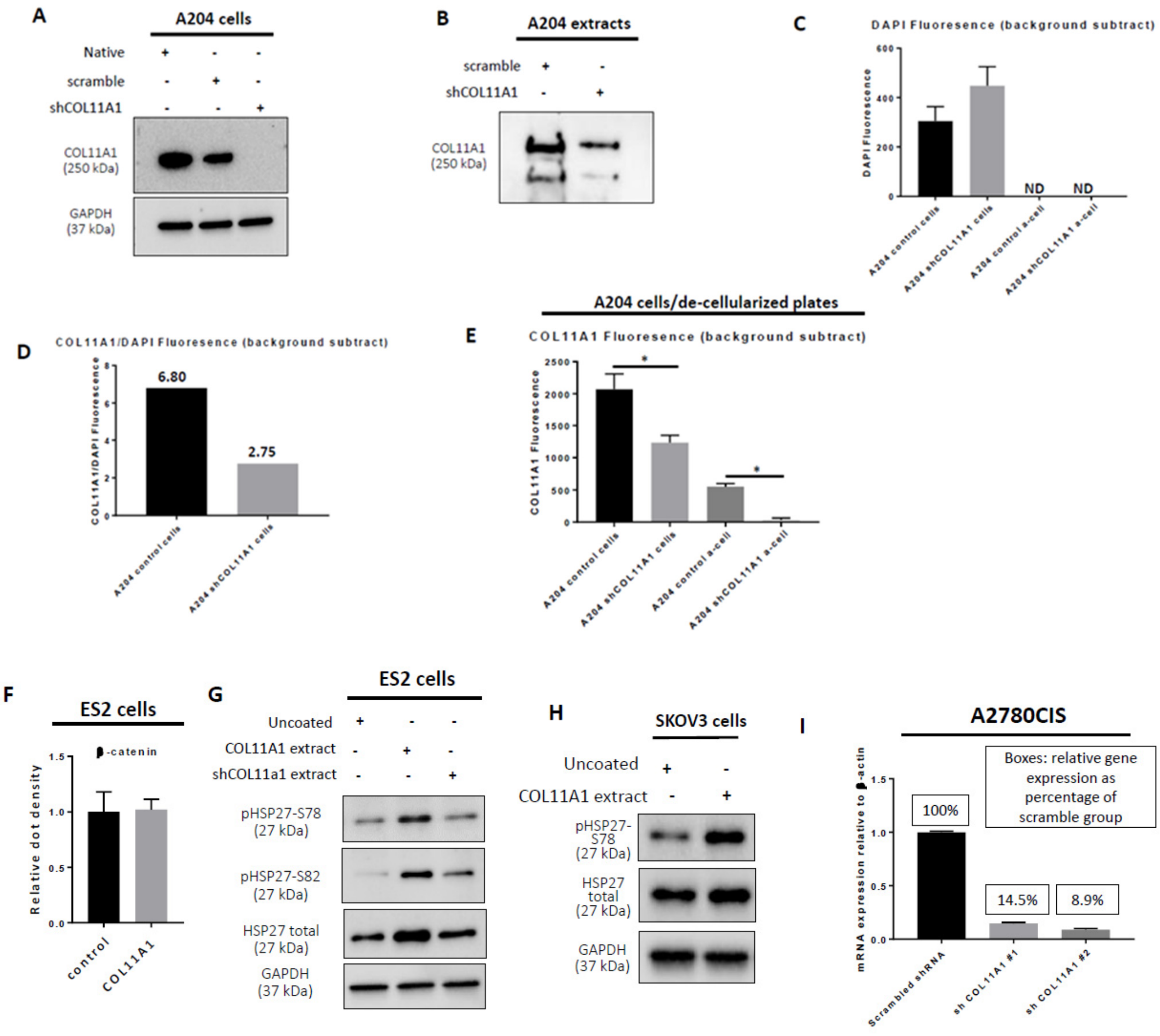

Figure S1. COL11A1 induces HSP27 phosphorylation and total expression in ovarian cancer cells. (**A**) Western blot of COL11A1 in native A204, A204-scr, and A204-shCOL11A1 cells collected from standard culture conditions (10% supplemented medium). (**B**) Western blot of COL11A1 in A204-scrm (COL11A1 positive) and A204-shCOL11A1 collagen extracts. (**C**) Immunofluorescence plate reader experiment showing average fluorescence of DAPI staining between A204-scrm and A204-shCOL11A1 cells and A204-scrm and A204-shCOL11A1 de-cellularized matrix groups. ND: Not detected. (**D**) Ratio of COL11A1 fluorescence (Alexa fluor 488 conjugated secondary antibody) compared to DAPI fluorescence in A204-scrm and A204-shCOL11A1 cells. (**E**) Plate reader

experiment showing average fluorescence of COL11A1 (Alexa fluor 488 secondary antibody) staining between A204-scrm and A204-shCOL11A1 cells and A204-scrm and A204-shCOL11A1 decellularized groups. (**F**) Quantification of β-catenin dot densities from PKA. (**G**) Western blot of phosphorylated (at S78 and S82) and total HSP27 in ES2 cells cultured on COL11A1 positive and negative A204 collagen extracts and control conditions. (**H**) Western blot of phosphorylated (at S78) and total HSP27 in SKOV3 cells cultured on COL11A1 positive and control conditions. (**I**) Gene expression of COL11A1 in A2780CIS-scrm and A2780CIS-shCOL11A1 #1 and #2 measured by RT-PCR. GAPDH was used as a loading control for western blots. All experiments performed in standard experiment conditions (cells cultured in above conditions for 3 days in 1% FBS medium after overnight serum starvation), unless otherwise noted. Error bars indicate standard deviation. *, p value <0.05. The uncropped Western blot figures are presented in Figure S10.

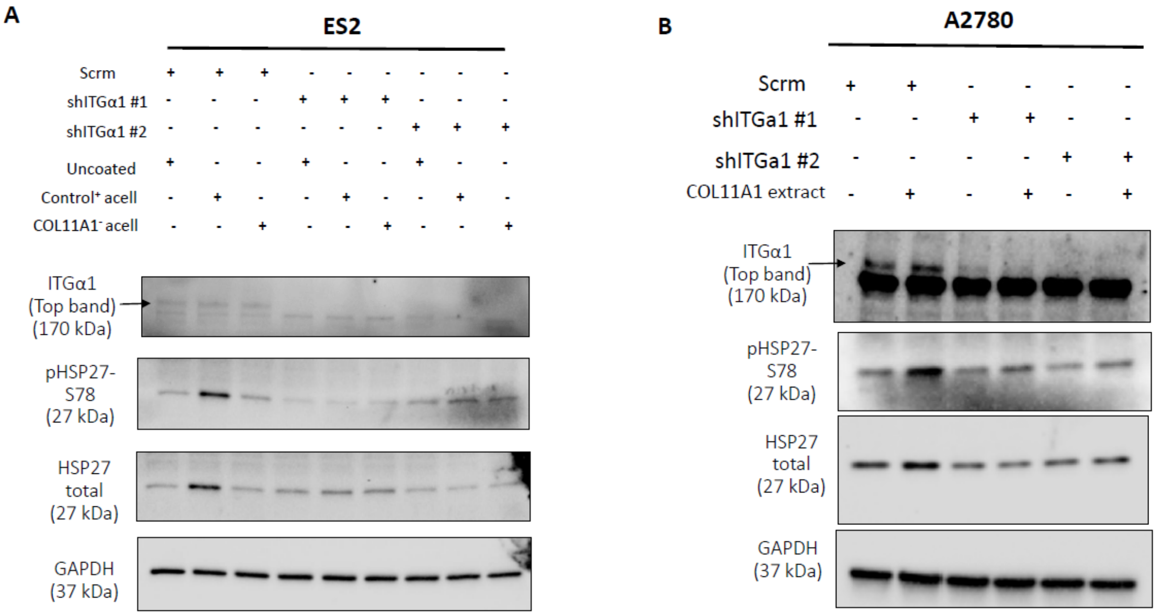

Figure S2. COL11A1 upregulates HSP27 phosphorylation and expression through activation of DDR2/integrin α1β1-Src-Akt signaling in ovarian cancer. (**A**) Western blot of ITGα1, phosphorylated (at S78) and total HSP27 in ES2-scrm and ES2-shITGα1 #1 and #2 cells. (**B**) Western blot of ITGα1, phosphorylated (at S78) and total HSP27 in A2780-scrm and A2780-shITGα1 #1 and #2 cells cultured on COL11A1 positive and control conditions. GAPDH was used as a loading control for western blots. All experiments performed in standard experiment conditions (cells cultured in above conditions for 3 days in 1% FBS medium after overnight serum starvation). The uncropped Western blot figures are presented in Figure S11.

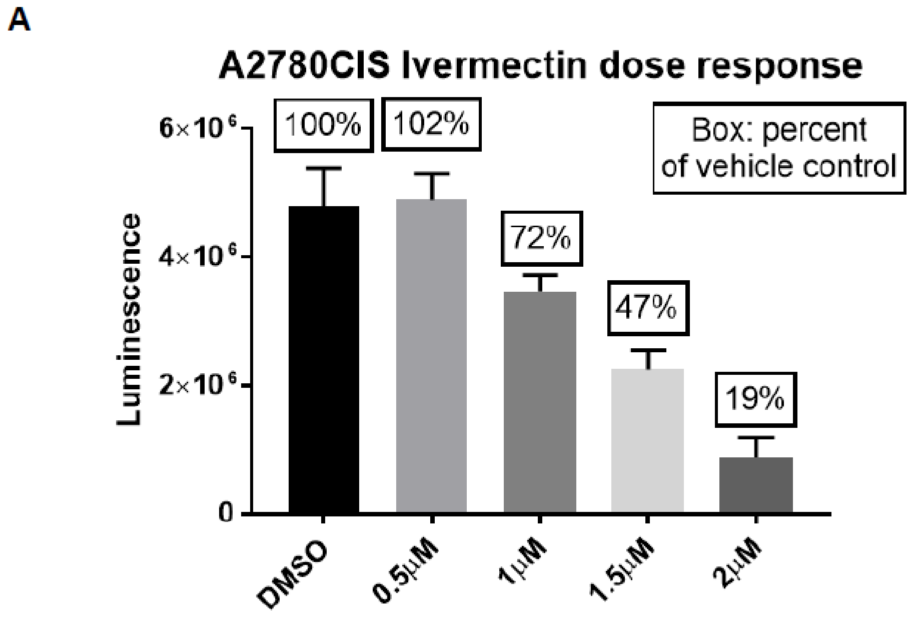

Figure S3. HSP27 mediates COL11A1-induced cisplatin resistance in ovarian cancer cells. (**A**) Relative cell viability (measured by Cell titer glo) of A2780CIS cells treated with 0.5μM, 1μM, 1.5μM. and 2μM Ivermectin (IVM) performed in standard experiment conditions (cells cultured in above conditions for 3 days in 1% FBS medium after overnight serum starvation).

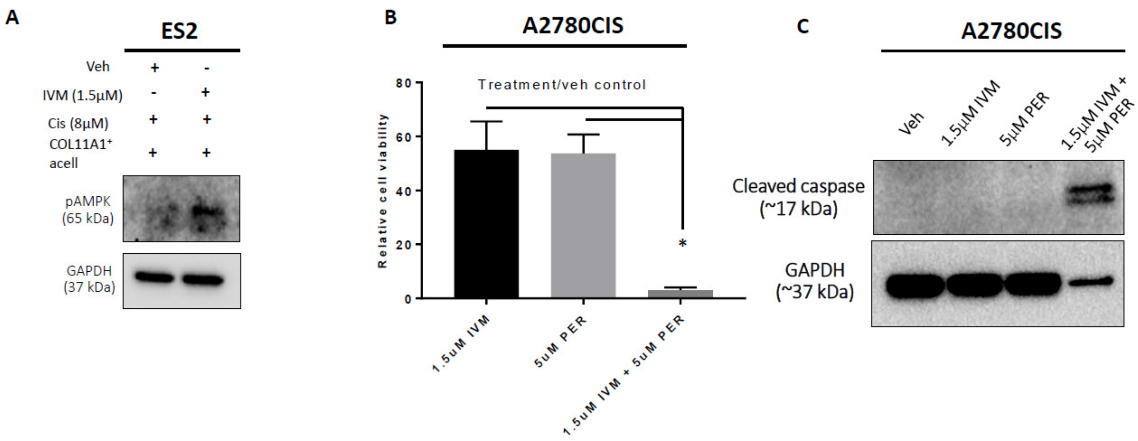

Figure S4. HSP27 synergizes with FAO to enhance cisplatin resistance. (**A**) Western blot of active (phosphorylated) AMPK in ES2 vehicle and 1.5μM ivermectin (IVM) treated cells treated with 8μM cisplatin (Cis) cultured on COL11A1 positive a-cellular matrices. (**B**) Relative cell viability (measured by acid phosphatase assay) of A2780CIS treated with 1.5μM ivermectin (IVM), 5μM perhexiline (PER), and combination treated 1.5μM ivermectin and 5μM perhexiline (IVM + PER) compared with vehicle treated control cells (n=3). (**C**) Western blot of cleaved caspase-3 in A2780CIS cells treated with 1.5μM ivermectin (IVM), 5μM perhexiline (PER), and combination treatment of 1.5μM ivermectin and 5μM perhexiline (IVM + PER). GAPDH was used as a loading control for western blots. All experiments performed in standard experiment conditions (cells cultured in above conditions for 3 days in 1% FBS medium after overnight serum starvation). Error bars indicate standard deviation. *, p value < 0.05. The uncropped Western blot figures are presented in Figure S12.

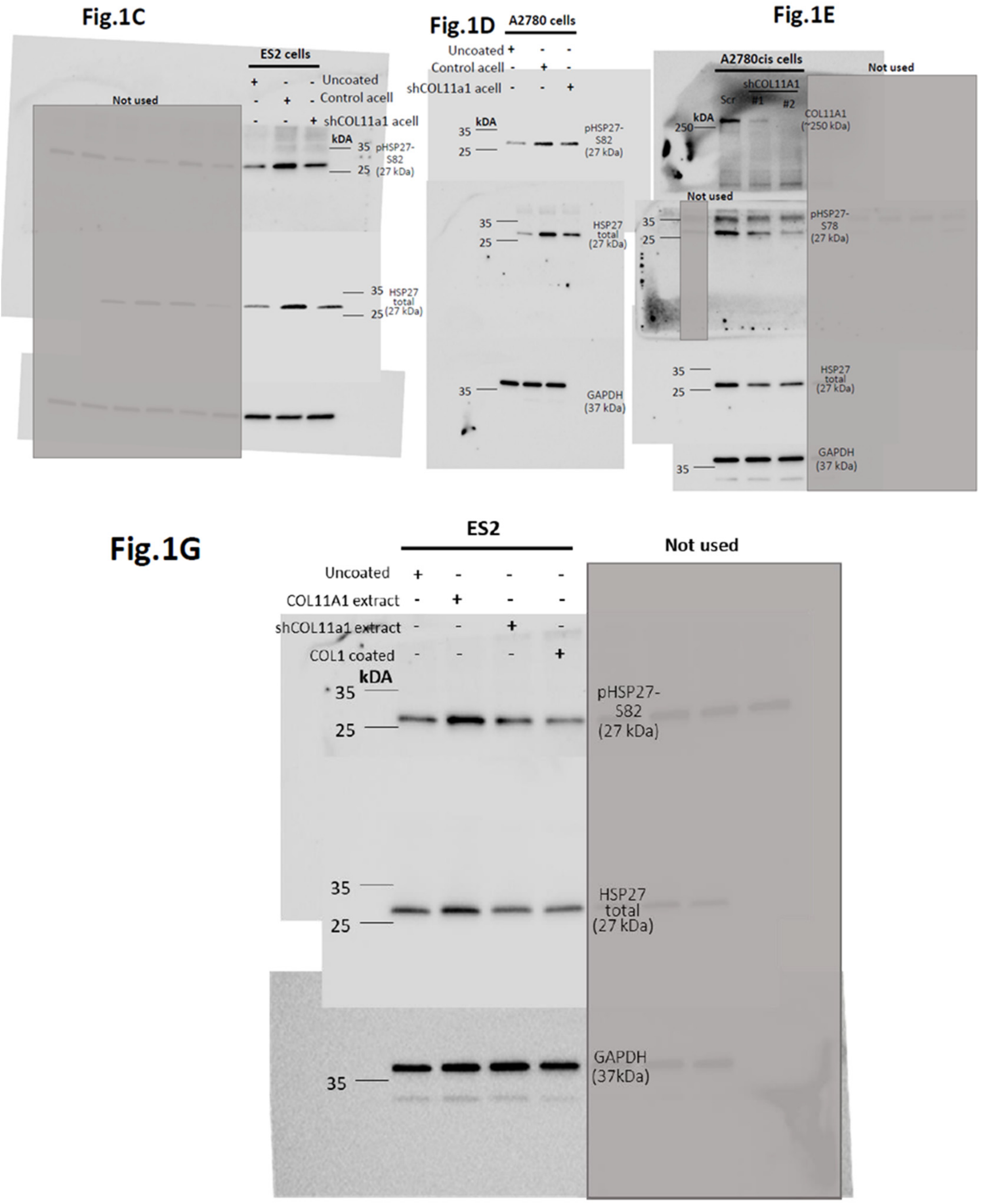

Figure S5. The uncropped western blot figures of Figure 1.

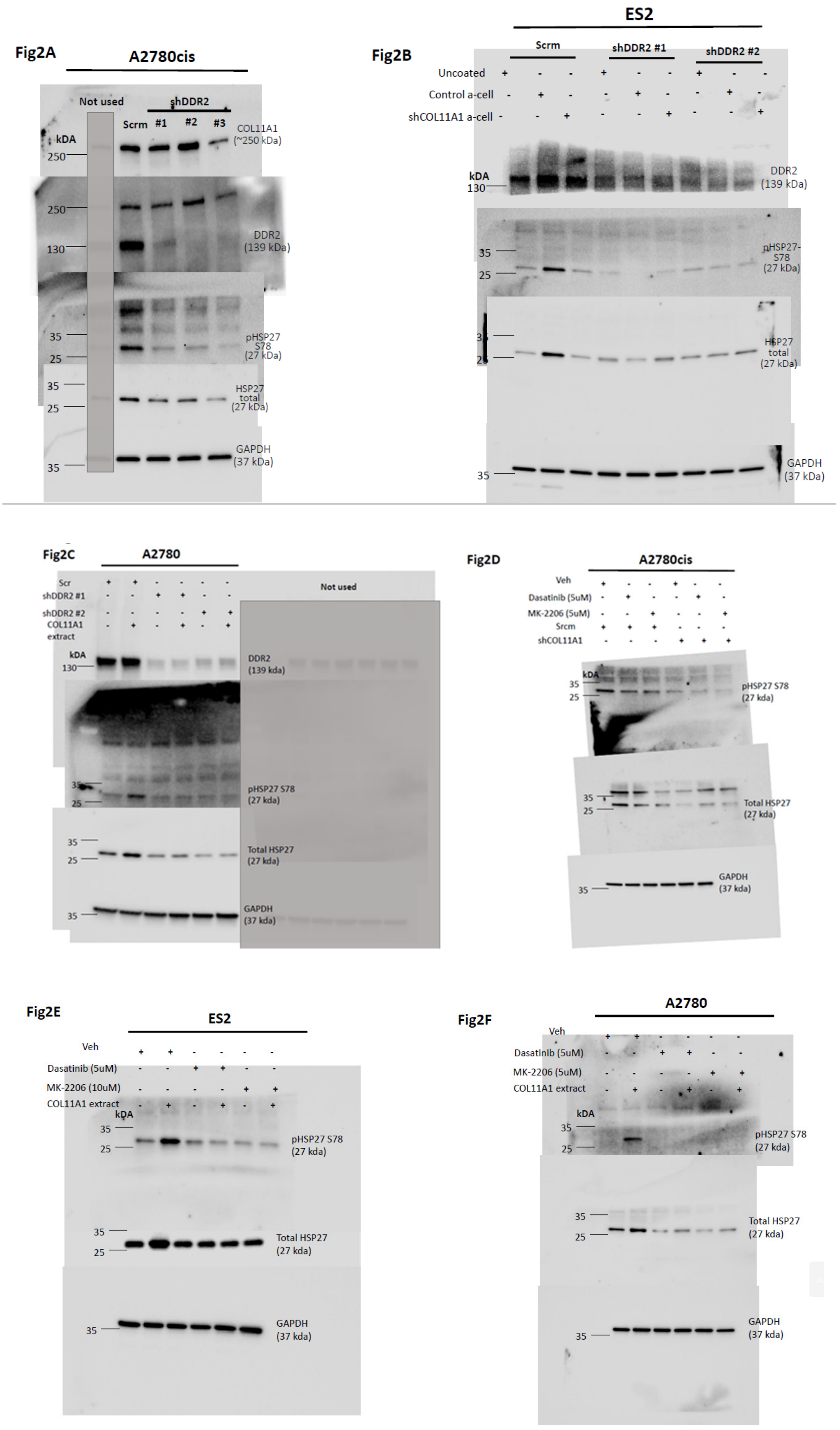

Figure S6. The uncropped western blot figures of Figure 2.

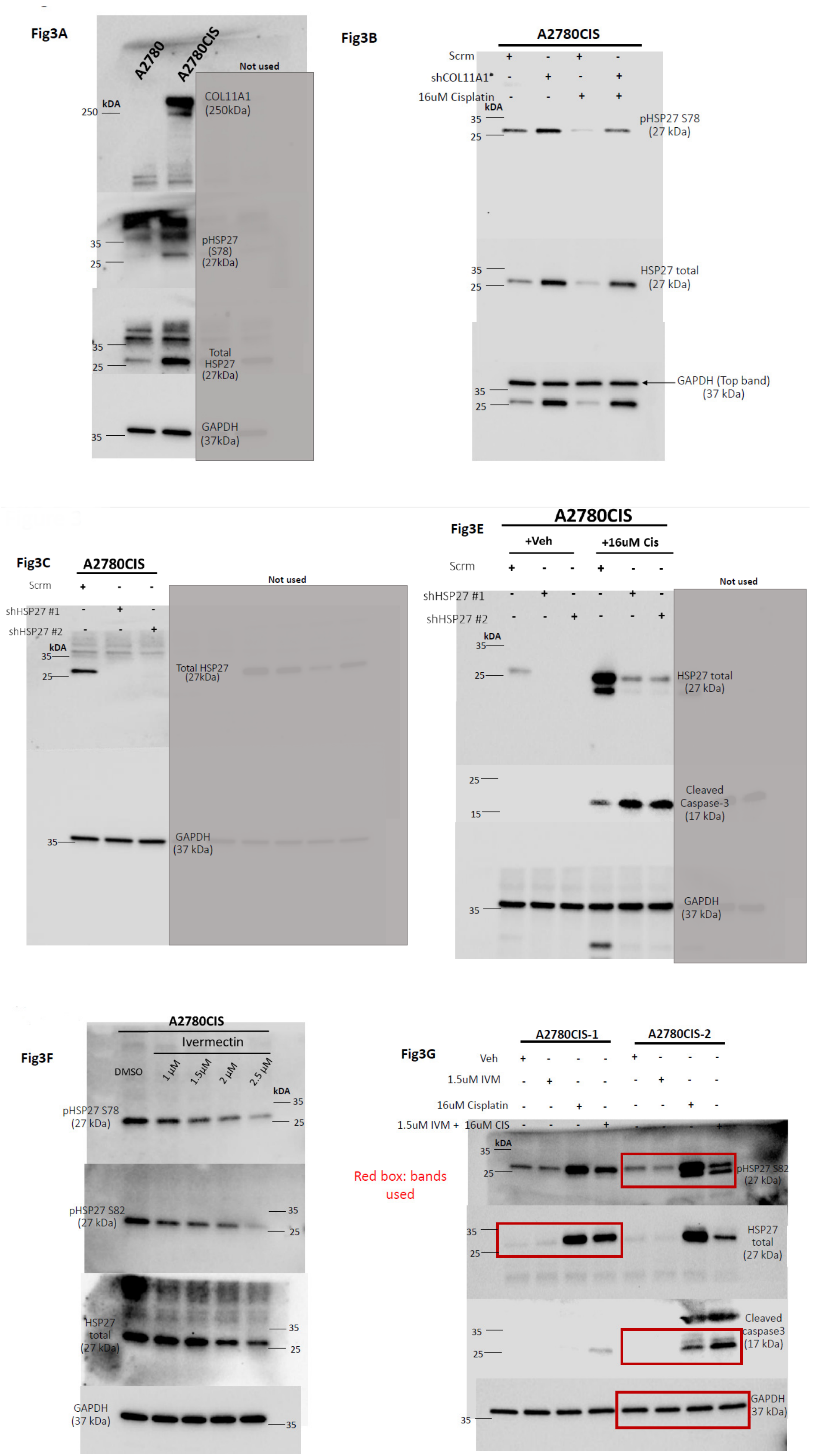
Fig3A
A2780
A2780CIS
Not used
kDA
250
COL11A1
(250kDa)
35
25
pHSP27
(S78)
(27kDa)
Total
HSP27
(27kDa)
GAPDH
(37kDa)
Fig3B
A2780CIS
Scrm
shCOL11A1*
16uM Cisplatin
pHSP27 S78
(27 kDa)
HSP27 total
(27 kDa)
GAPDH (Top band)
(37 kDa)
Fig3C
A2780CIS
Scrm
shHSP27 #1
shHSP27 #2
Not used
Total HSP27
(27kDa)
GAPDH
(37 kDa)
Fig3E
A2780CIS
+Veh
+16uM Cis
Scrm
shHSP27 #1
shHSP27 #2
Not used
HSP27 total
(27 kDa)
Cleaved
Caspase-3
(17 kDa)
GAPDH
(37 kDa)
Fig3F
A2780CIS
Ivermectin
DMSO
1 μM
1.5uM
2 μM
2.5 μM
pHSP27 S78
(27 kDa)
pHSP27 S82
(27 kDa)
HSP27
total
(27 kDa)
GAPDH
(37 kDa)
Fig3G
A2780CIS-1
A2780CIS-2
Veh
1.5uM IVM
16uM Cisplatin
1.5uM IVM + 16uM CIS
Red box: bands
used
pHSP27 S82
(27 kDa)
HSP27
total
(27 kDa)
Cleaved
caspase3
(17 kDa)
GAPDH
37 kDa)

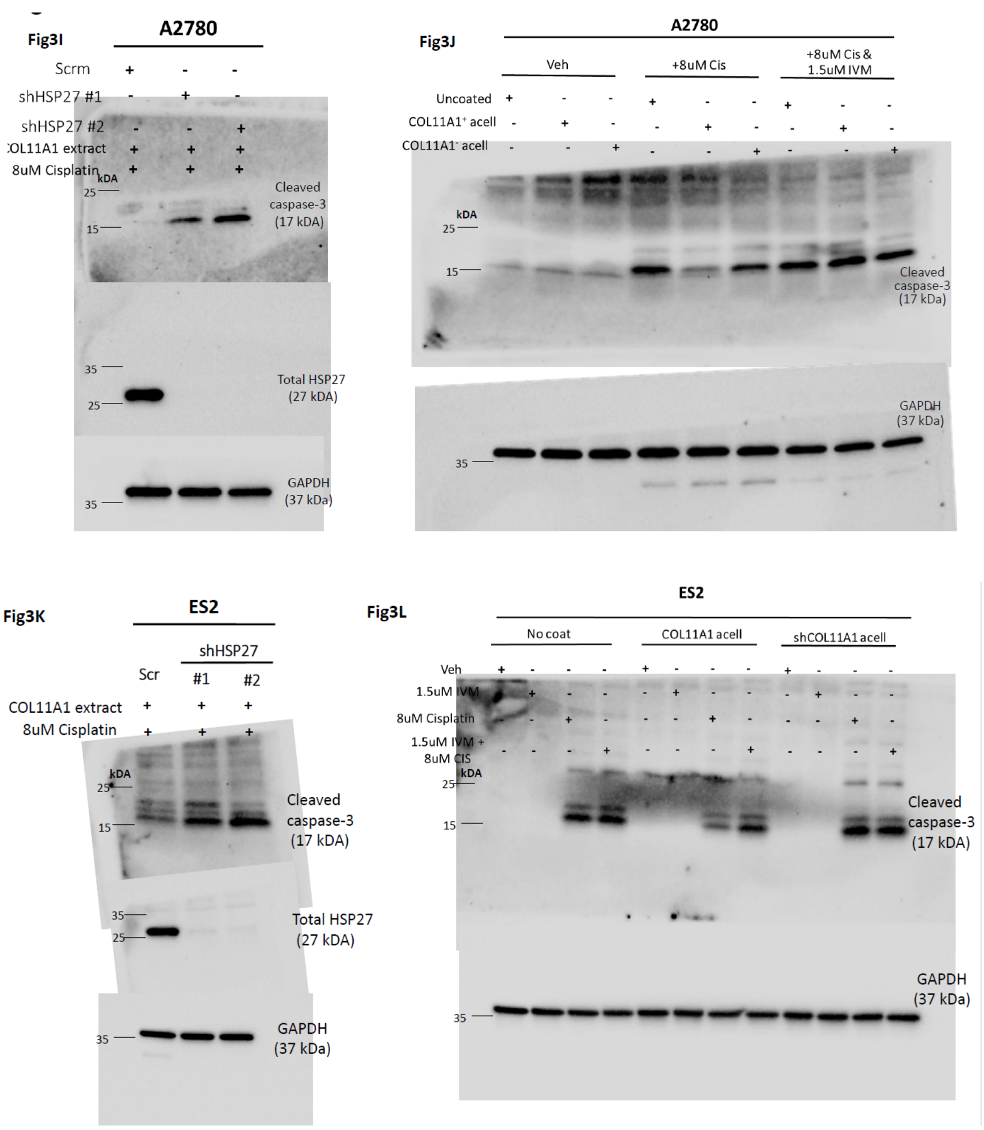

Figure S7. The uncropped western blot figures of Figure 3.

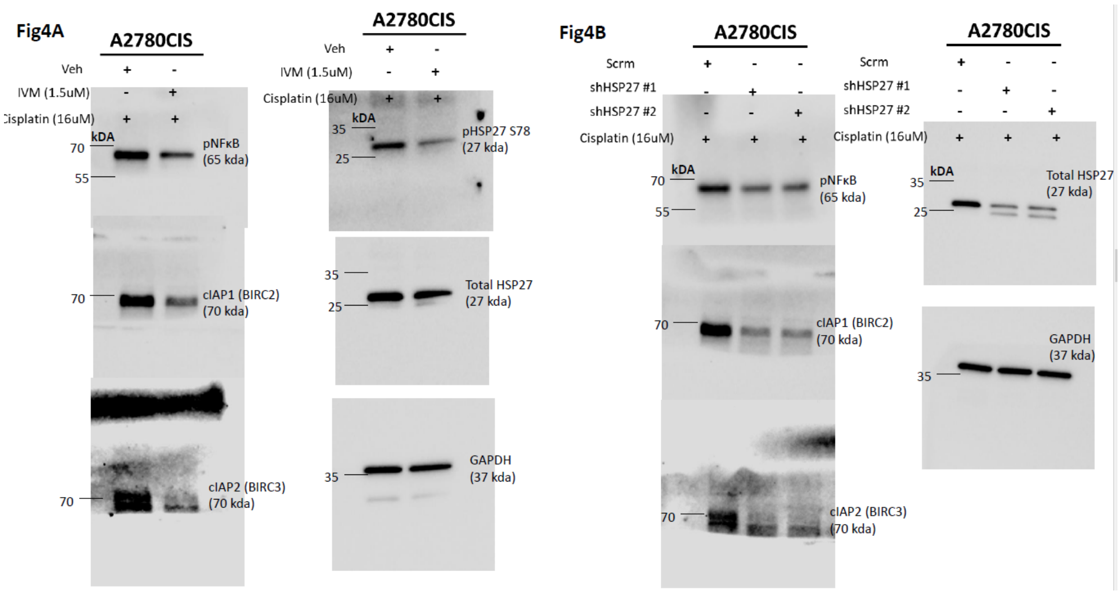

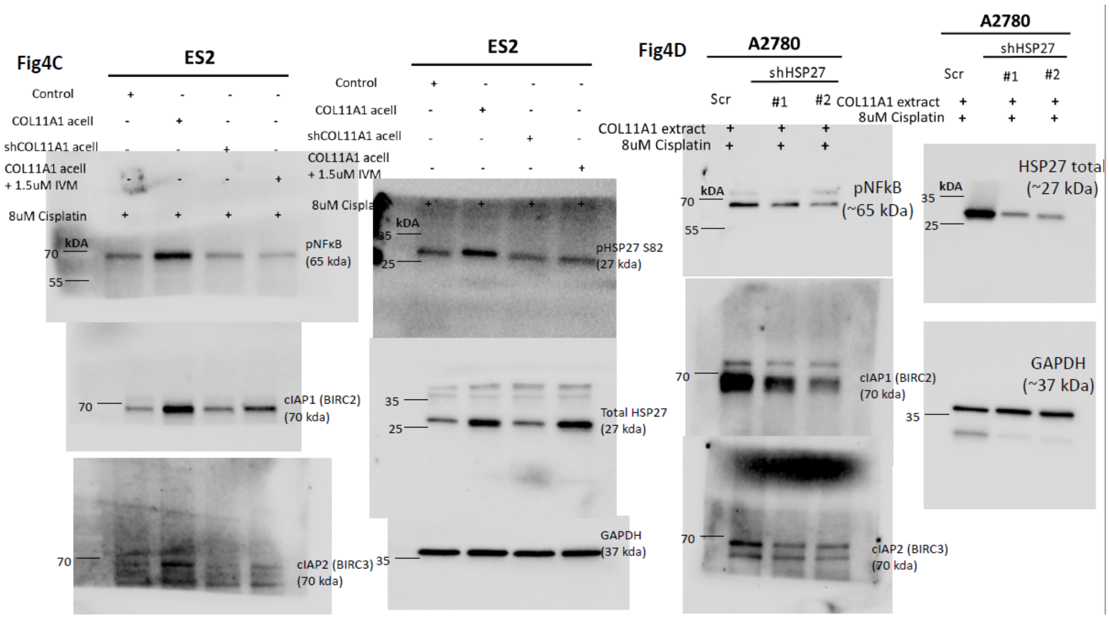

Figure S8. The uncropped western blot figures of Figure 4.

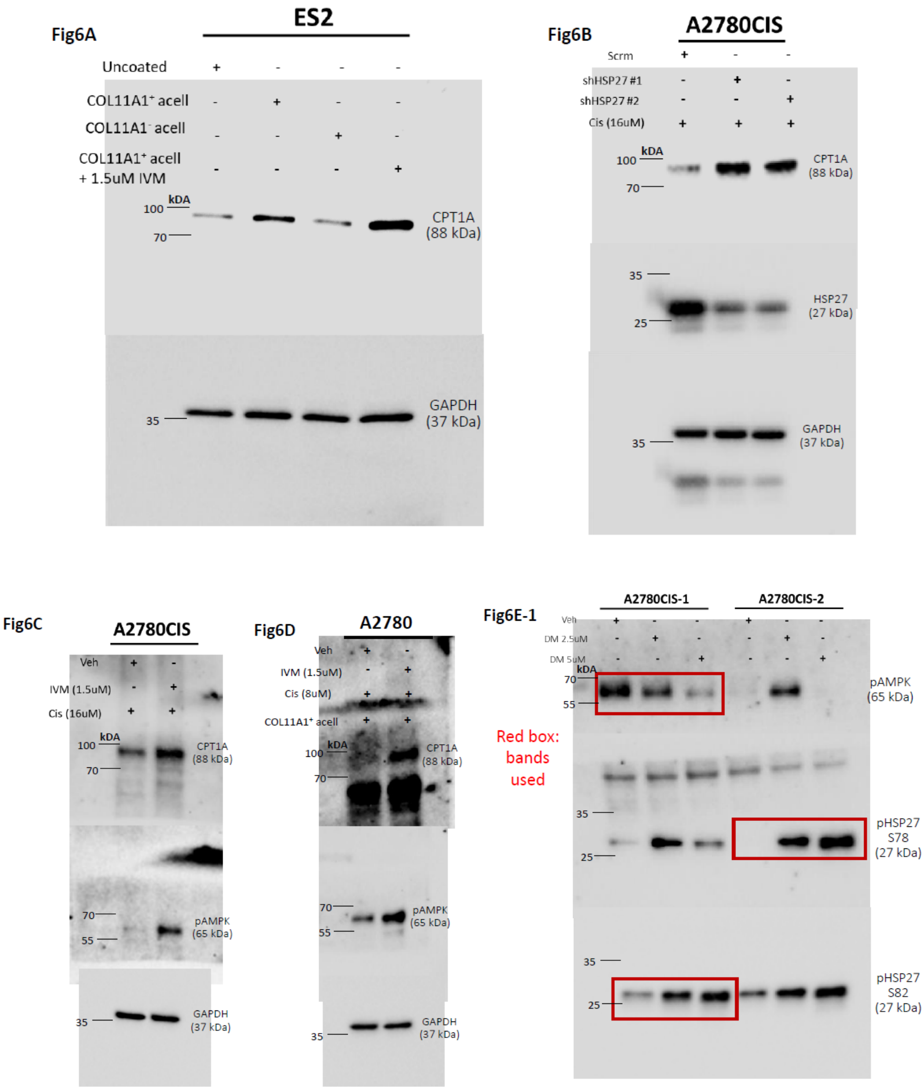

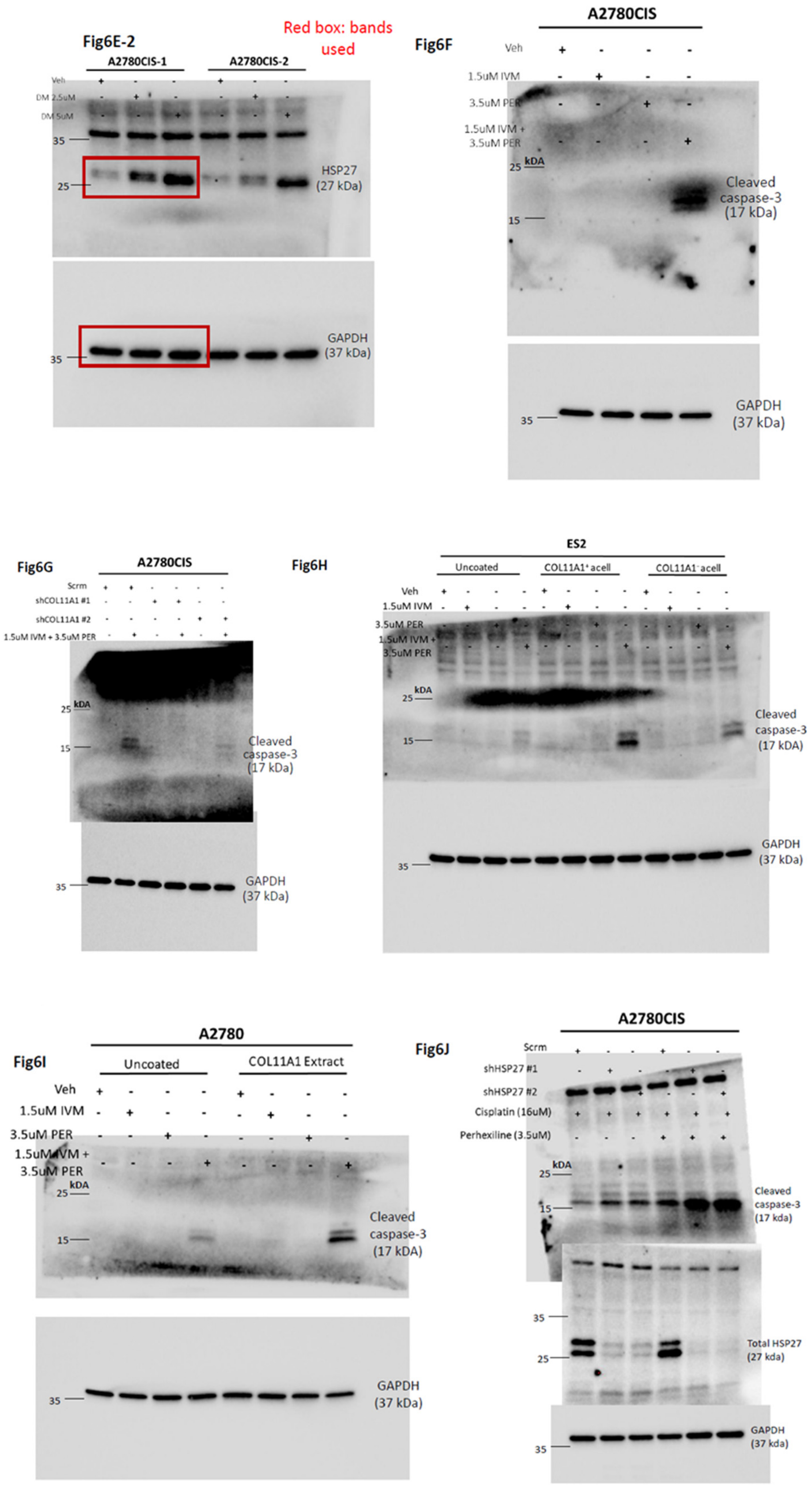

Figure S9. The uncropped western blot figures of Figure 6.

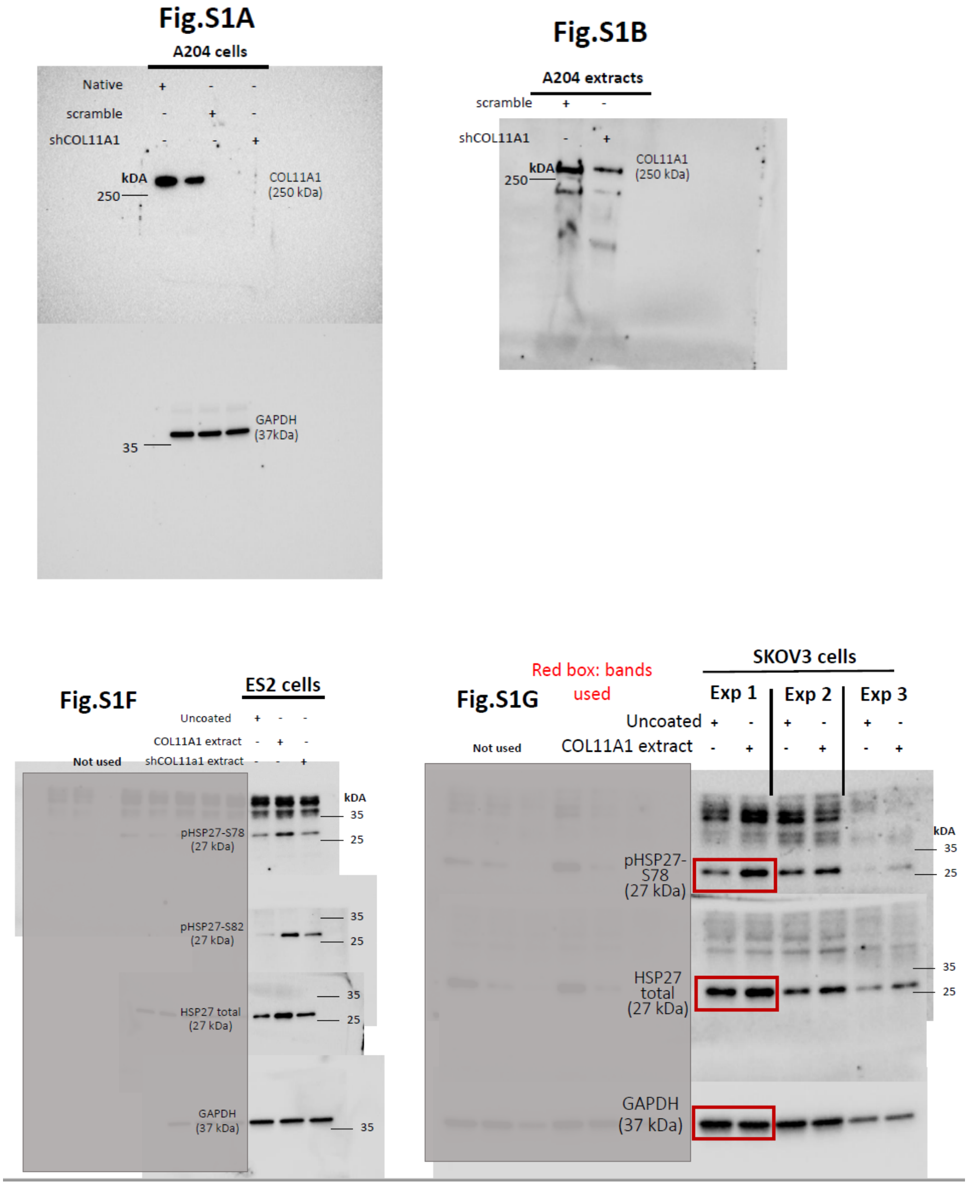

Figure S10. The uncropped western blot figures of Figure S1.

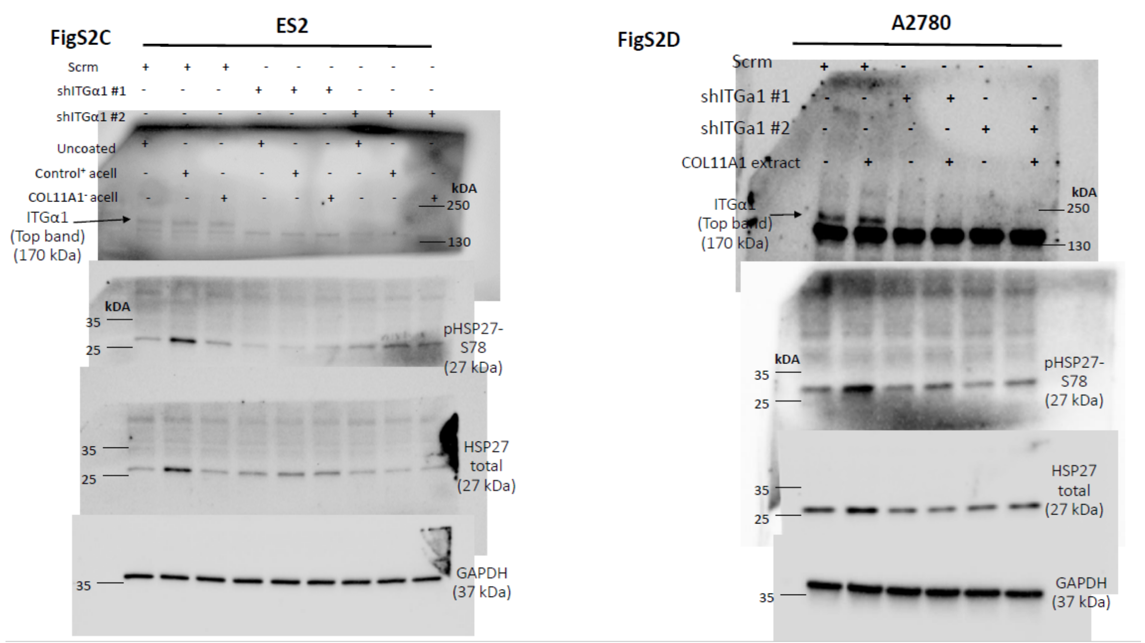

Figure S11. The uncropped western blot figures of Figure S2.

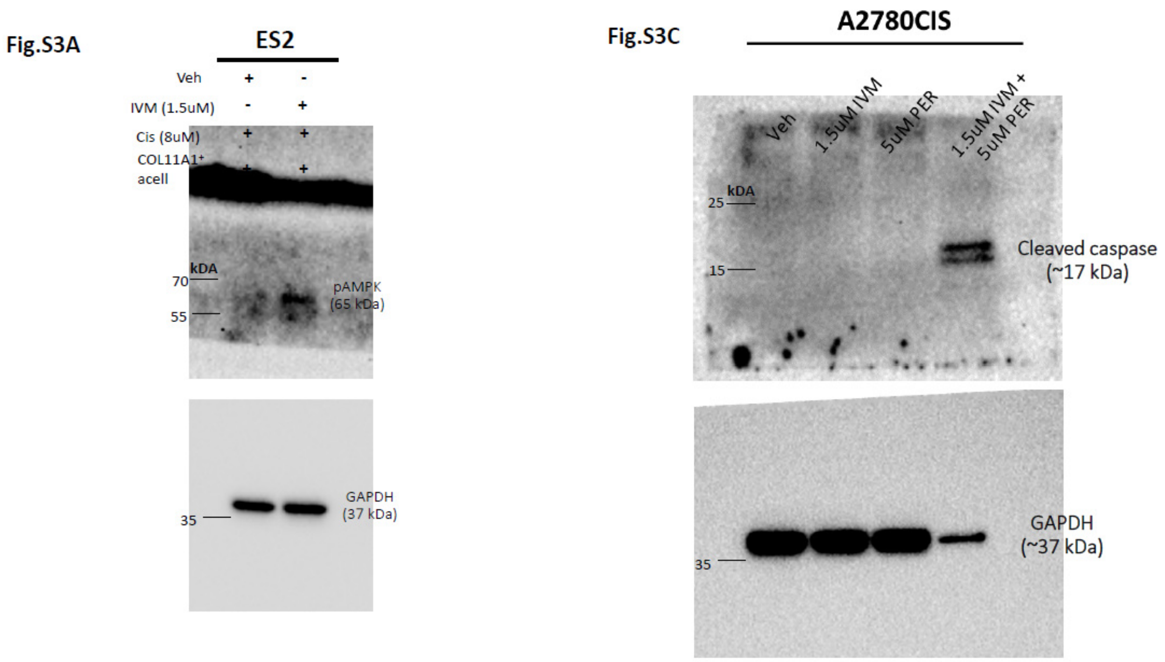

Figure S12. The uncropped western blot figures of Figure S3.

Table S1. Lentiviral constructs used in this study.

Plasmid construct/ mission shRNA plasmid DNA	Product #	Company/Source
pCMV-ΔR 8.2 dvpr	#8455 Addgene	Gift (Dr. Weinberg Lab)
pCMV-VSVG	#8454 Addgene	Gift (Dr. Weinberg Lab)
scrambled vector	#SHC016	Sigma-Aldrich
shCOL11A1 #1 and #2	#TRCN0000083377<br>#TRCN0000083373	Sigma-Aldrich
shDDR2 #1 #2 and #3	#TRCN0000199773<br>#TRCN0000121262	Sigma-Aldrich

	#TRCN0000196465	
shITGα1 #1 and #2	#HSH009793-LVRU6H-a<br>#HSH009793-LVRU6H-d	GeneCopoeia
shHSPB1 #1 #2 #3 and #4	#TRCN0000008754<br>#TRCN0000342857<br>#TRCN0000342790<br>#TRCN0000356587	Sigma-Aldrich

Table S2. Real-time PCR primer sequences used in this study.

Gene	Primer sequence
HSPB1	F: 5′- CTGACGGTCAAGACCAAGGATG-3′<br>R: 5′- GTGTATTTCCGCGTGAAGCACC-3′
HSPB8	F: 5′- CAGAGGAGTTGATGGTGAAGACC-3′<br>R: 5′- ACTGTCACAGGATCCACCTCTG-3′
HSPB11	F: 5′- AGCCAGTTGATTTTGAGCAATGGA-3′<br>R: 5′- GCAGAAACGCTATGCACAGATGC -3′
HSP60	F: 5′- TGCCAATGCTCACCGTAAGCCT-3′<br>R: 5′- AGCCTTGACTGCCACAACCTGA -3′
HSP70	F: 5′- GACCTGCCAATCGAGAATCAGC-3′<br>R: 5′- CTGCGTTCTTAGCATCATTCCGC-3′
RPL32	F: 5′-ACAAAGCACATGCTGCCCAGTG-3′<br>R: 5′-TTCCACGAT-GGCTTTGCGGTTC-3′
GAPDH	F: 5′- ACAGTCAGCCGCATCTTCTT-3′<br>R: 5′- TTGATTTTGGAGGGATCTCG-3′
β-actin	F: 5′- GCTGTGCTACGTCGCCCTG-3′<br>R: 5′- GGAGGAGCTGGAAGCAGCC-3′

Table S3. List of antibodies, kit and reagents used in this study.

Antibody, kit or reagent	Product #	Company
Human Phospho-Kinase Array kit	ARY003B	R&D Systems
BIRC2 (antibody)	#sc-271419	Santa Cruz Biotechnology
BIRC3 (antibody)	#sc-7944	Santa Cruz Biotechnology
Phospho-HSP27 (Ser78) (antibody)	#2405	Cell Signaling Technology
Phospho-HSP27 (Ser82) (antibody)	#9709	Cell Signaling Technology
HSP27 (antibody)	#2402	Cell Signaling Technology
Cleaved caspase-3 (antibody)	#9664P	Cell Signaling Technology
pNF-kB (S536) (antibody)	#3033P	Cell Signaling Technology
COL11A1 (antibody)	#ab64883	Abcam
DDR2 (antibody)	#Y740	R&D Systems
pAMPK (T172) (antibody)	#2535S	Cell Signaling Technology
GAPDH (antibody)	#10R-G109a	Fitzgerald
Goat-anti rabbit secondary (antibody), FITC conjugated	N/A	Gift from Dr. Gang Liu
Alexafluor 488 conjugated secondary (antibody) (anti-rabbit)	#A-11034	Invitrogen
Alexafluor 488 conjugated secondary (antibody) (anti-mouse)	#A-11029	Invitrogen
Goat-anti mouse secondary (antibody)	#170-6516	Bio-Rad
Goat-anti rabbit secondary (antibody)	#170-6515	Bio-Rad
Donkey-anti goat secondary (antibody)	#ab97110	abcam
DAPI	#D1306	Invitrogen
Type I collagen	#5005	Advanced BioMatrix

Cisplatin	#P4394	Sigma Aldrich
Ivermectin	#I8898-25mg	Sigma Aldrich
Dorsomorphin	#AB144821	Abcam
Dasatinib	#73082	Stem Cell technologies
MK-2206	#A3010	ApexBio

Table S4. Top ten differentially regulated proteins from Protein Kinase array.

Protein (Phosphorylated residue)	Fold change (COL11A1/No Coat control, normalized to β-catenin)
CREB (S133)	2.17
HSP27 (S78/82)	1.74
eNOS (S1177)	1.62
ERK1/2 (T202/Y204, T185/Y187)	1.59
Src (Y419)	1.50
Akt (S473)	1.41
AMPKα1 (T183)	1.40
c-Jun (S63)	1.35
GSK3α/β (S21, S19)	1.16
STAT3 (S727)	−1.63
